# High‐Plex Digital Spatial Profiling Identified Prolactin‐Induced Protein mRNA Associated With Response and Survival of Everolimus and Letrozole Treatment for Hormone Receptor‐Positive/Human Epidermal Growth Factor Receptor 2‐Negative Advanced Breast Cancer

**DOI:** 10.1002/mco2.70509

**Published:** 2025-11-26

**Authors:** Yuhang Han, Danyang Ji, Yujing Tan, Jiayu Wang, Fei Ma, Yang Luo, Bo Lan, Pin Zhang, Jianming Ying, Binghe Xu, Liyan Xue, Ying Fan

**Affiliations:** ^1^ Department of Medical Oncology National Cancer Center/National Clinical Research Center for Cancer/Cancer Hospital Chinese Academy of Medical Sciences and Peking Union Medical College Beijing China; ^2^ Department of Pathology National Cancer Center/National Clinical Research Center for Cancer/Cancer Hospital Chinese Academy of Medical Sciences and Peking Union Medical College Beijing China

**Keywords:** high‐plex digital spatial profiling, HR+/HER2− advanced breast cancer, everolimus, letrozole, PIP mRNA

## Abstract

Everolimus (EVE) combined with letrozole is an approved treatment for hormone receptor‐positive/human epidermal growth factor receptor 2‐negative (HR+/HER2−) advanced breast cancer (ABC). However, predictive biomarkers for EVE efficacy remain undefined. In the phase 2 MIRACLE trial, we performed digital spatial profiling (DSP) on pretreatment tumor samples from 21 patients receiving EVE plus letrozole. Patients were divided into resistant and sensitive groups based on their best response to EVE. A total of 119 regions across three compartments—tumor, leukocytes, and stroma—were profiled for immune and transcriptomic markers. Responders had significantly higher fibroblast infiltration in PANCK+ (*p* = 0.011) and CD45−/PANCK− (*p* = 0.043) regions, whereas non‐responders exhibited increased neutrophils in CD45+ (*p* = 0.0061) and PANCK+ (*p* = 0.03) regions. Prolactin‐induced protein (PIP) mRNA expression was significantly elevated in non‐responders in both PANCK+ (*p* < 0.0001) and CD45−/PANCK− (*p* = 0.0006) regions. PIP mRNA expression was found to be associated with EVE resistance and unfavorable progression‐free survival (PFS). PIP mRNA expression and specific immune‐stromal features are associated with resistance to EVE. These findings suggest the potential of PIP as a spatially resolved predictive biomarker for patient stratification in HR+/HER2− ABC.

## Graphical abstract

In the phase 2 MIRACLE trial, we performed digital spatial profiling (DSP) on pretreatment tumor samples from 21 patients receiving EVE plus letrozole. Patients were divided into resistant and sensitive groups based on their best response to EVE. Responders had significantly higher fibroblast infiltration in PANCK+ and CD45−/PANCK− regions, whereas non‐responders exhibited increased neutrophils in CD45+ and PANCK+ regions. Prolactin‐induced protein (PIP) mRNA expression was found to be associated with EVE resistance and unfavorable progression‐free survival (PFS).

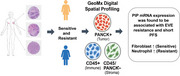

## 1 Introduction

Hormone receptor‐positive/human epidermal growth factor receptor 2‐negative (HR+/HER2−) breast cancer represents approximately 60% of all breast cancer cases and is generally responsive to endocrine therapy (ET), leading to favorable clinical outcomes [1, 2]. The current first‐line treatment for patients with HR+/HER2− advanced breast cancer (ABC) involves the use of cyclin‐dependent kinase 4/6 (CDK4/6) inhibitors in combination with ET [3, 4, 5, 6]. However, therapeutic resistance tends to emerge over time, and the optimal strategy for patients who progress after CDK4/6 inhibitor therapy remains unclear. Current options include switching to an alternative CDK4/6 inhibitor plus ET, chemotherapy, ET monotherapy, or targeted therapy such as everolimus (EVE).

EVE, a mammalian target of rapamycin (mTOR) inhibitor, is one of the most extensively studied strategies for HR+/HER2− ABC [7]. The BOLERO‐2 trial demonstrated that EVE combined with exemestane significantly prolonged progression‐free survival (PFS) versus exemestane alone in postmenopausal patients [8, 9, 10]. In our MIRACLE trial, a prospective phase 2 study in premenopausal patients with HR+/HER2− ABC, EVE, plus letrozole significantly prolonged median PFS compared with letrozole alone (19.4 vs. 12.9 months) [11]. However, clinical benefit remains variable, some patients derive durable responses, and others experience early disease progression. Notably, approximately 10% of patients in BOLERO‐2 progressed rapidly after starting EVE [8], and this proportion was even higher in MIRACLE trial, reaching 27.3% [11]. These observations underscore the need for effective biomarkers to identify patients most likely to benefit from EVE and avoid unnecessary costs and toxicity.

Several studies have attempted to correlate EVE efficacy with gene mutational profiles such as mutations in PIK3CA or other PAM pathway components [12, 13, 14]. However, results have been inconclusive, and no robust predictive biomarkers have been identified. Given the complex biology of mTOR signaling, attention has shifted toward evaluating the tumor microenvironment (TME) and its components. The mTOR signaling pathway plays a critical role in immune regulation by integrating diverse environmental inputs in the TME, involving CD8+ and CD4+ lymphocytes, regulatory T cells (Tregs), macrophages, myeloid‐derived suppressor cells, endothelial cells, and tumor‐associated fibroblasts [15, 16, 17]. Inhibition of the mTOR pathway may influence anti‐tumor immunity, malignant progression, and tumor response. One study has indicated that dysregulation of the TME may be involved in resistance to mTOR inhibitors in non‐breast cancer patients [18], but its relevance in HR+/HER2− breast cancer remains insufficiently explored.

Recent advances in spatial transcriptomic technologies have enabled high‐resolution profiling of gene expression and immune architecture within specific tissue compartments. Digital spatial profiling (DSP) is a powerful tool because it analyzes mRNA from specific parts of formalin‐fixed paraffin‐embedded (FFPE) specimens, which allows DSP to find heterogeneity within a tumor, and it could be key to demonstrating how patients respond to treatment [19].

In this study, we applied DSP to analyze pretreatment tumor samples from patients enrolled in the EVE plus letrozole arm of the MIRACLE phase 2 trial (NCT02313051). By assessing spatial mRNA expression and immune infiltration across tumor, leukocyte, and stromal compartments, we sought to identify transcriptomic and microenvironmental features associated with EVE response and survival outcomes. These findings are intended to provide insights into the spatial biology of therapeutic resistance in HR+/HER2− ABC.

## 2 Results

### 2.1 DSP of HR+/HER2− ABC Patients Treated With EVE‐Combined Therapy

Twenty‐two patients from the EVE arm of the MIRACLE trial were screened based on hematoxylin and eosin (HE)‐stained slides. However, one patient was excluded due to the lack of specific cells in two tumor cores, leaving 21 patients for further analysis.

**FIGURE 1:**
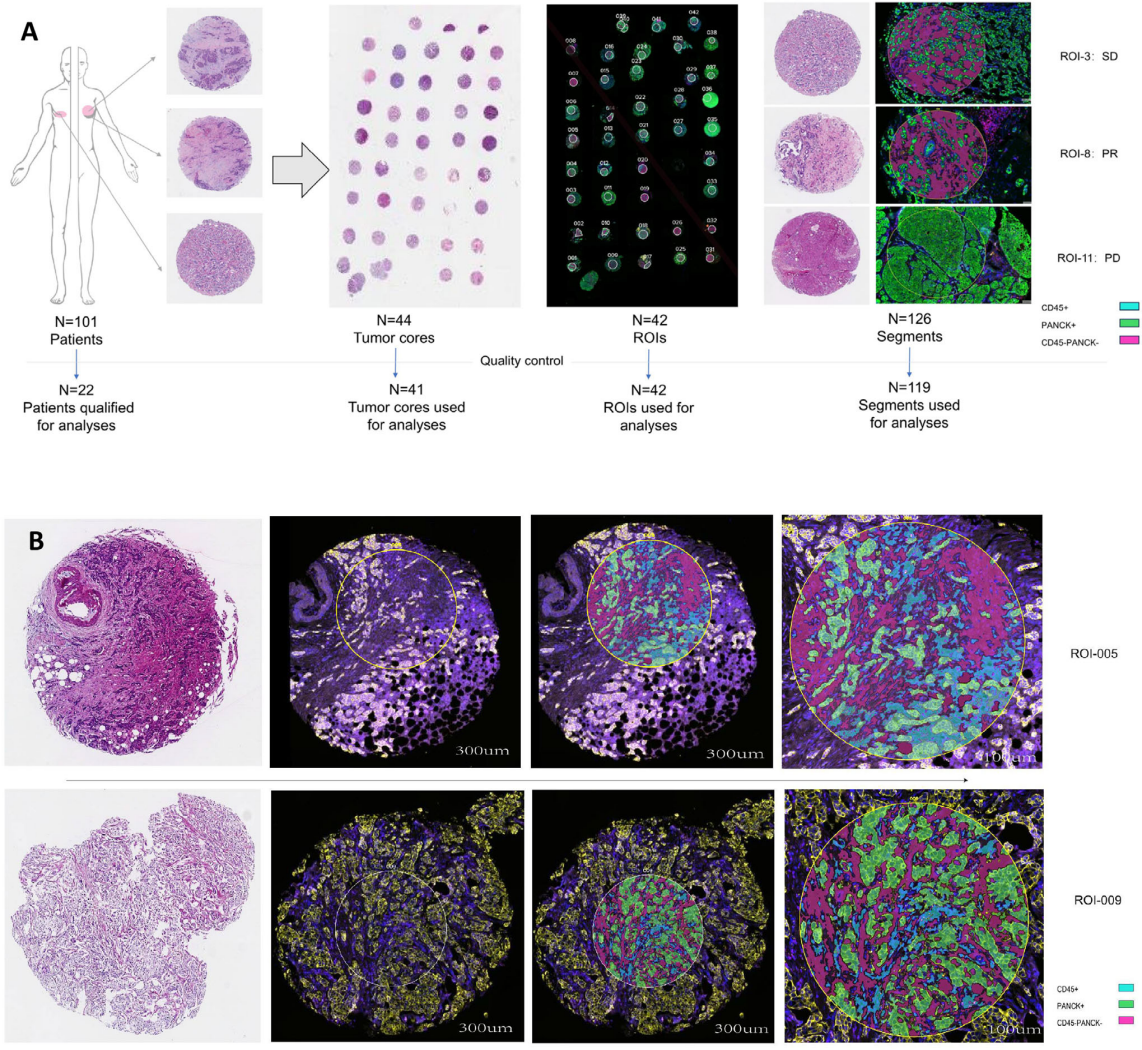
Preparation of sections for DSP analysis. (A) Schematic of patient enrollment and DSP analysis in the study. PD: progressive disease, PR: partial response, SD: stable disease, ROI: region of interest. (B) Representative tissue microarray spots showing the hematoxylin and eosin staining images (left) and fluorescence images (right). Fluorescence images showing the segments created by visualization antibodies using GeoMx DSP (scale bar, 100 µm). The blue represents the CD45+ zone, the green represents the PANCK+ tumor zone, and the rose red represents the CD45−/PANCK− zone. The area within the circle represents one ROI. ROI‐005 was obtained from a patient who achieved PR to EVE and had a PFS of 73.8 months, whereas ROI‐009 was derived from a patient who experienced SD to EVE and had a PFS of 26.2 months. ROI distribution was as follows: CD45+ zones, SD+PR (*n* = 33) and PD (*n* = 8); PANCK+ zones, SD+PR (*n* = 32) and PD (*n* = 6); CD45−/PANCK− zones, SD+PR (*n* = 32) and PD (*n* = 8).

Two tumor cores from FFPE tissues of each patient were collected to construct the tissue microarray (TMA) block. In one case, one core was disqualified due to tissue floating during fluorescence colocalization. Consequently, two nonadjacent regions of interest (ROIs) (denoted as 039 and 040) were selected from the remaining tumor core. In the results, 42 ROIs were available for mathematical segmentation (Figure 1A, Figure S1). Each ROI was divided into three areas of interest (AOIs) based on fluorescent‐labeled visualization antibodies, including PANCK+ (tumor cell cluster), CD45+ (immune cell cluster), and CD45−/PANCK− (stromal cell cluster) regions (Figure 1B). In theory, this yielded 126 zones across all ROIs. However, seven zones were excluded during quality control (QC) due to insufficient representation of one or more compartments. Ultimately, 119 spatially defined zones and two negative controls were included for RNA analysis using DSP. Details on ROI composition are provided in Table S1 and Figure S1.

Patients were categorized into two groups based on their best response to EVE plus letrozole. Four patients who experienced progressive disease (PD) were classified as EVE resistant. Seven patients with stable disease (SD) lasting over 6 months and 10 patients with partial response (PR) were classified as EVE sensitive. Clinicopathological characteristics of both groups are presented in Table 1. Notably, the EVE‐resistant group had a higher prevalence of histological grade (*p* = 0.0012) and significantly more visceral‐only metastases (75% vs. 11.8%, *p* = 0.04). Other baseline characteristics were generally similar between groups.

**TABLE 1:** Baseline characteristics of patients qualified for DSP analyses.

Characteristics	EVE‐sensitive group (*N* =17)	EVE‐resistant group (*N* = 4)	Total (*N* = 21)	*p* value
Age				0.12
Mean ± SD	42.2 ± 6.5	50.0 ± 3.6	43.4 ± 6.8	
Median [min‐max]	45.0 (32.0, 49.0)	51.0 (45.0, 53.0)	46.0 (32.0, 53.0)	
Histological grade^a^				1.20E‐03
I	1 (5.9%)	0 (0.0e+0%)	1 (5.3%)	
II	14 (82.4%)	1 (25.0%)	15 (78.9%)	
III	0 (0.0e+0%)	3 (75.0%)	3 (15.8%)	
Histology type				
IDC	17 (100%)	4 (100%)	21 (100%)	
ECOG performance				0.56
0	5 (29.4%)	0 (0.0e+0%)	5 (23.8%)	
1	12 (70.6%)	4 (100%)	16 (76.2%)	
T stage				1
1	11 (64.7%)	3 (75.0%)	14 (66.7%)	
2	6 (35.3%)	1 (25.0%)	7 (33.3%)	
N stage				1
0	4 (23.5%)	1 (25.0%)	5 (23.8%)	
1	5 (29.4%)	2 (50.0%)	7 (33.3%)	
2	5 (29.4%)	1 (25.0%)	6 (28.6%)	
3	3 (17.6%)	0 (0.0e+0%)	3 (14.3%)	
M stage				1
0	16 (94.1%)	4 (100%)	20 (95.2%)	
1	1 (5.9%)	0 (0.0e+0%)	1 (4.8%)	
Position				1
Right	7 (33.33%)	2 (9.52%)	9 (42.86%)	
Left	10 (47.62%)	2 (9.52%)	12 (57.14%)	
Surgery^b^				0.53
Breast conserving	3 (14.29%)	0 (0.0e+0%)	3 (14.29%)	
Modified radical mastectomy	12 (57.14%)	3 (14.29%)	15 (71.43%)	
Neoadjuvant therapy	4 (23.5%)	1 (25.0%)	5 (23.8%)	1
Adjuvant therapy				
Endocrine therapy	17 (100%)	4 (100%)	21 (100.00%)	
Radiotherapy	11 (64.7%)	1 (25.0%)	12 (57.1%)	0.38
Chemotherapy	12 (70.6%)	4 (100%)	16 (76.2%)	0.56
Metastatic sites for the first relapse				
Lymph node	4 (23.5%)	0 (0.0e+0%)	4 (19.1%)	0.71
Lung	4 (23.5%)	0 (0.0e+0%)	4 (19.1%)	0.71
Liver	2 (11.8%)	3 (75.0%)	5 (23.8%)	0.04
Bone	12 (70.6%)	1 (25.0%)	13 (61.9%)	0.26
Bone‐only	9 (52.9%)	1 (25.0%)	10 (47.6%)	0.65
Visceral‐only	2 (11.8%)	3 (75.0%)	5 (23.8%)	0.04
Number of metastatic sites for the first relapse				0.43
One	11 (64.7%)	4 (100%)	15 (71.4%)	
More than one	6 (35.3%)	0 (0.0e+0%)	6 (28.6%)	

Abbreviations: ECOG, Eastern Cooperative Oncology Group; IDC, invasive ductal carcinoma; SD, standard deviation.

^a^ Information of pathological grade from two EVE‐sensitive patients was missing.

^b^ The operative approach was unknown in one EVE‐sensitive patient and one EVE‐resistant patient. One EVE‐sensitive patient who was initially diagnosed with stage IV disease underwent palliative mastectomy.

**FIGURE 2:**
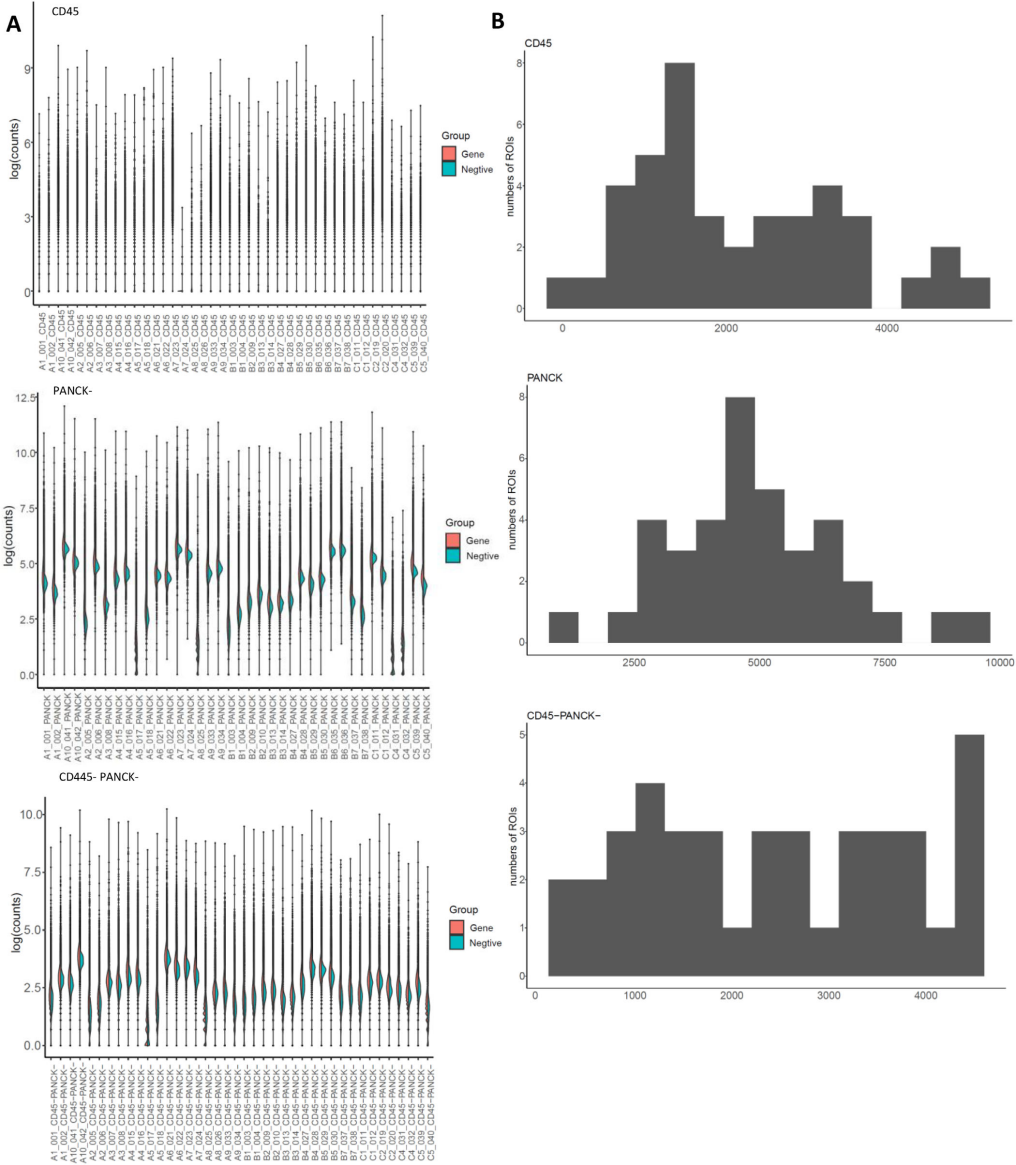
Quality control assessment of DSP RNA assay performance. (A) Split violin plots overlaid with scatterplots showing RNA assay counts across participants. Gene counts are shown on the right side (red) and the geometric mean of negative probe counts is shown on the left side (green). Zones are arranged from top to bottom as the CD45+ zone, the PANCK+ zone, and the CD45−/PANCK− zone. (B) Box plots showing the number of targets (genes) based on the limit of quantitation (LOQ) per ROI in the spatially defined regions for the DSP RNA assay. The equation for LOQ is presented as follows: *LOQ = GeoMean(NegProbe)×GeoSD(NegProbe)* ^2^.

Gene expression was quantified using Illumina next‐generation sequencing (NGS). Probe counts were quality‐filtered by removing outliers and computing geometric means per gene. Distributions of gene and negative probe counts for each sample across CD45+, PANCK+, and CD45−/PANCK− zones are shown in Figure 2A. The limit of quantification (LOQ) was defined by the formula [LOQ = GeoMean(NegProbe) × GeoSD(NegProbe)^2^]. The median number of genes above LOQ was 1968 in CD45+ zones (range: 59–5205), 4628 in PANCK+ zones (range: 897–9,108), and 2488 for CD45−/PANCK− zones (range: 325–4516) (Figure 2B, Figure S2). These results confirmed adequate RNA quantity and quality across spatial compartments, supporting the feasibility of downstream DSP analyses.

### 2.2 Immune Cell Landscape in Spatially Resolved Regions of HR+/HER2− ABC Patients With Different Treatment Responses to EVE‐Combined Therapy

**FIGURE 3:**
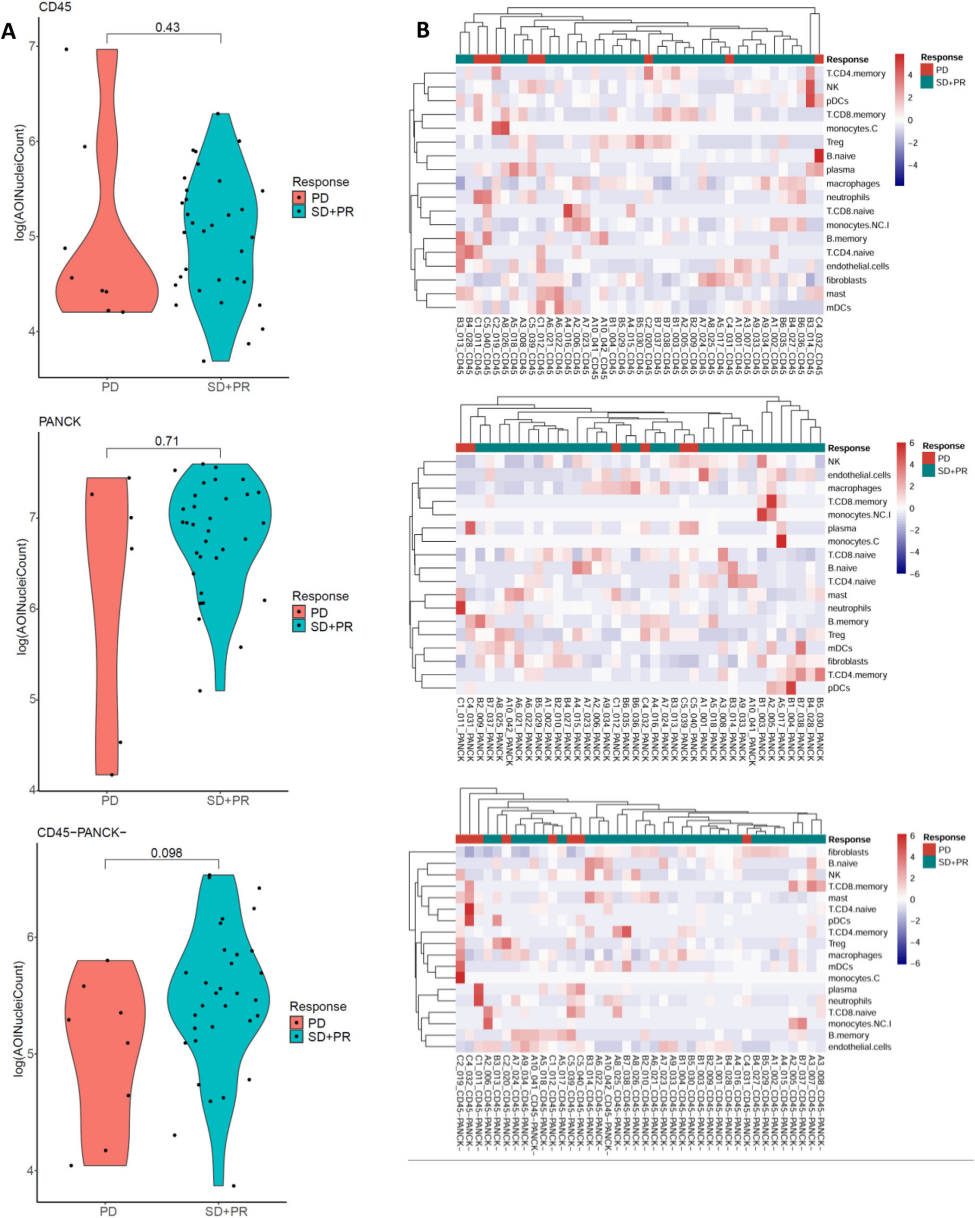
Immune cell infiltration analysis in spatially defined regions. (A) Violin plots showing TME‐related cell aggregates in patients who responded to EVE (SD+PR) versus those with PD. Sample size (*n*) indicates the number of regions of interest (ROIs). (B) Heatmap showing infiltration of 18 types of immune cells in the two groups.

Using the DSP system, we analyzed the TME in patients with HR+/HER2− ABC who responded differently to EVE plus letrozole. No significant differences were observed in the total number of TME‐related cells between the EVE‐sensitive and EVE‐resistant groups across the CD45+ zone (*p* = 0.43), the PANCK+ zone (*p* = 0.71), and the CD45−/PANCK− zone (*p* = 0.098) (Figure 3A).

**FIGURE 4:**
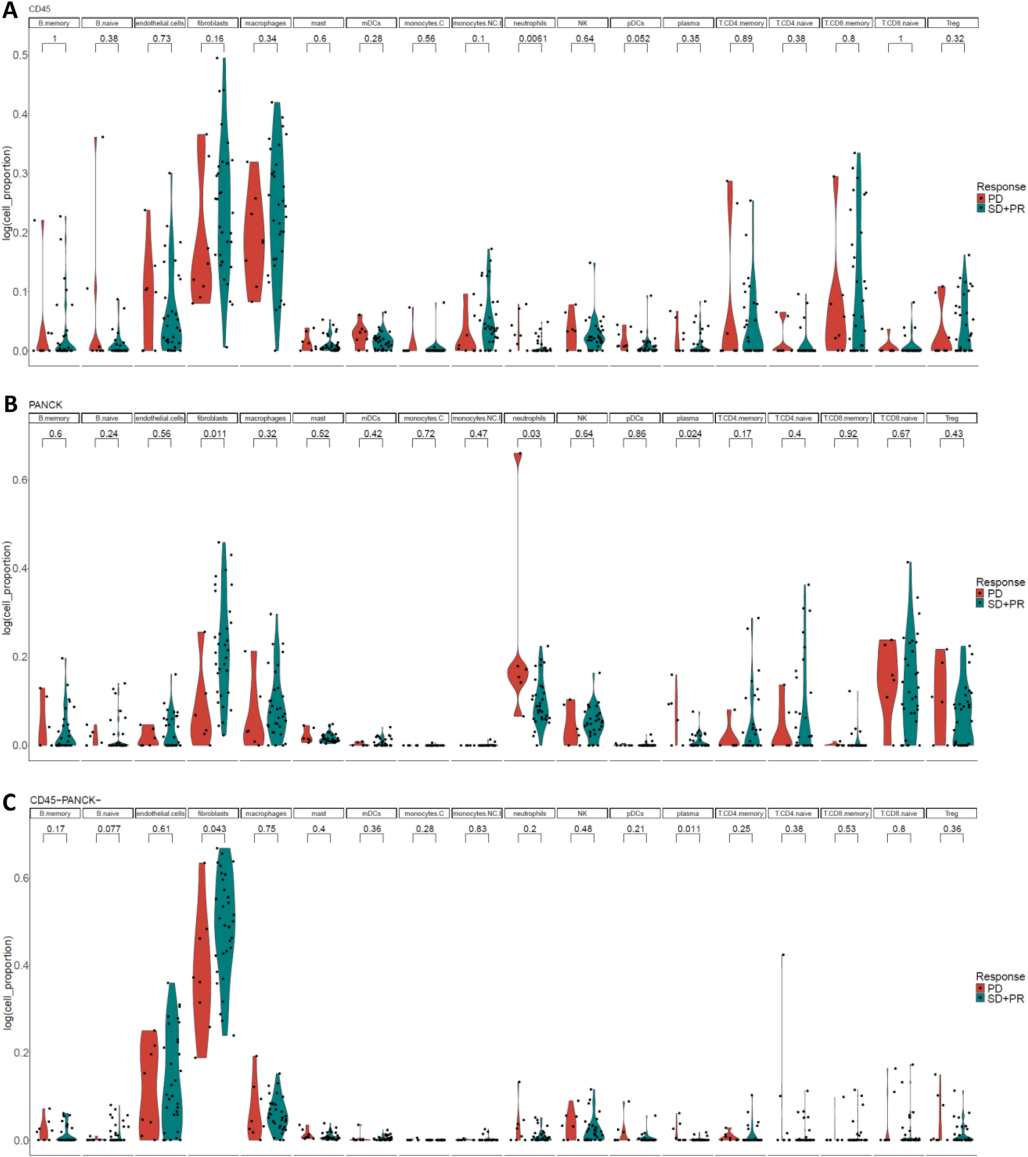
Violin plots displaying cell proportion of 18 TME‐related cells for patients with (SD+PR) versus PD. *p* value was displayed. (A) CD45+ zones. (B) PANCK+ zones. (C) CD45−/PANCK− zones.

SpatialDecon analysis was applied to quantify 18 TME‐related cell types across three spatial zones (Figure 3B). Figure 4 summarizes differences in the infiltration levels of the 18 TME‐related cell types between the EVE‐sensitive and EVE‐resistant groups.

Among the 41 CD45+ zones, macrophages and fibroblasts were the dominant cell types (median proportions: 24.6% and 22.1%, respectively). Neutrophil infiltration was significantly higher in the EVE‐resistant group (*p* = 0.0061). Other immune cell types did not show significant variation (Figure 4A).

In the 38 PANCK+ tumor regions, fibroblasts had the highest proportion (median 21.0%), especially in EVE‐sensitive patients (23.6% vs. 5.3%, *p* = 0.011). Plasma cell infiltration was significantly higher in the resistant group (5.9% vs. 0%, *p* = 0.024), as was neutrophil infiltration (17.9% vs. 8.8%, *p* = 0.03). CD8+ T naive cells were the second most abundant cell type but did not differ significantly between groups (Figure 4B).

The CD45−/PANCK− zones were dominated by fibroblasts and endothelial cells, accounting for over 70% of total cells. Fibroblast proportions were significantly higher in the EVE‐sensitive group (64.1% vs. 44.2%, *p* = 0.043), while plasma cells were more abundant in resistant tumors (0.2% vs. 0%, *p* = 0.011) (Figure 4C).

### 2.3 Quantitative Transcriptomic Measurements of Spatially Resolved Regions to Identify Differentially Expressed Genes

**FIGURE 5:**
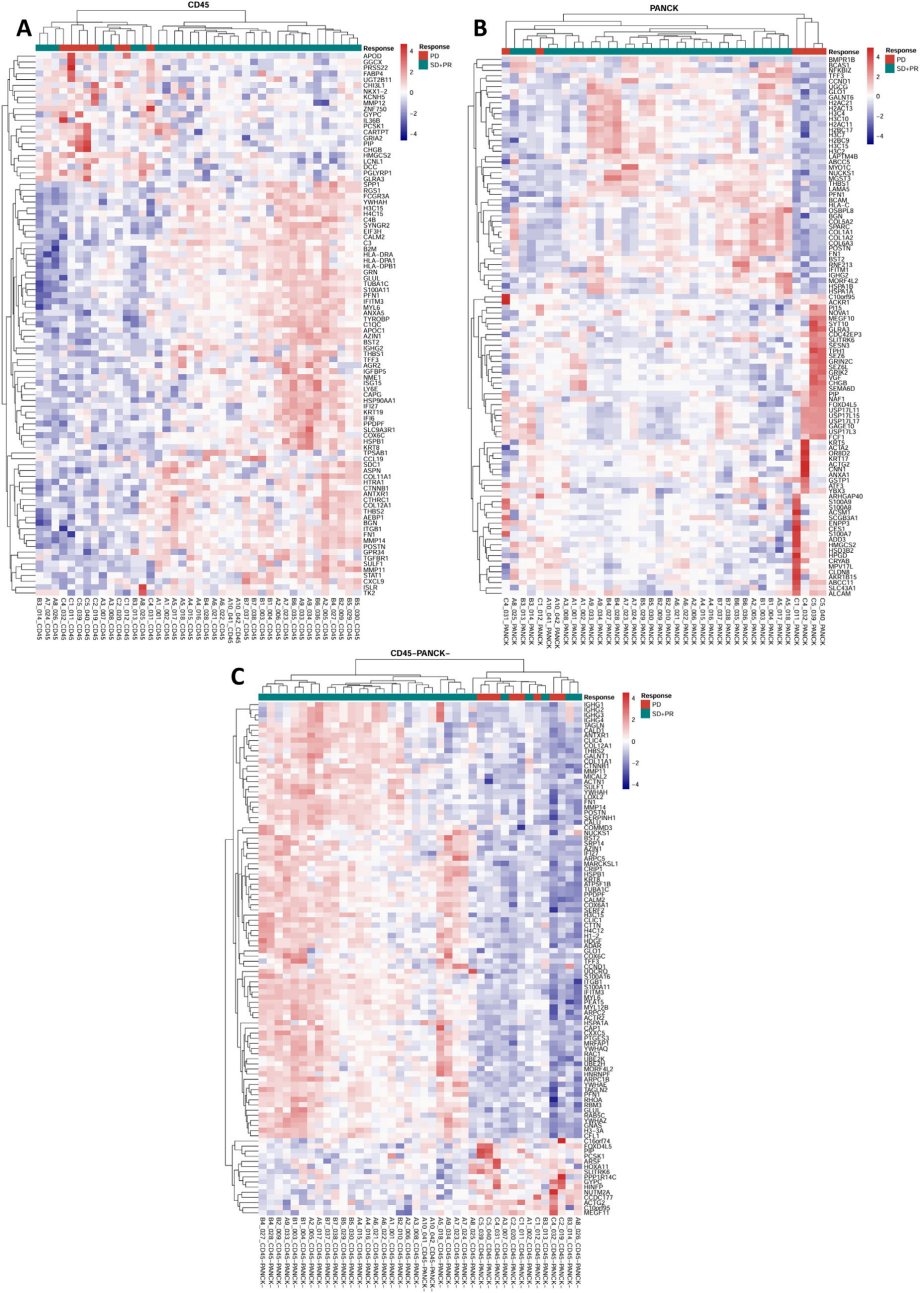
Heatmap of DEGs between the EVE‐sensitive and EVE‐resistant groups. Note that 93 DEGs in the CD45+ zones and the top 100 DEGs ranked by significance in the PANCK+ zones and the CD45‐/PANCK‐ zones were presented. (A) CD45+ zones. (B) PANCK+ zones. (C) CD45−/PANCK− zones.

**FIGURE 6:**
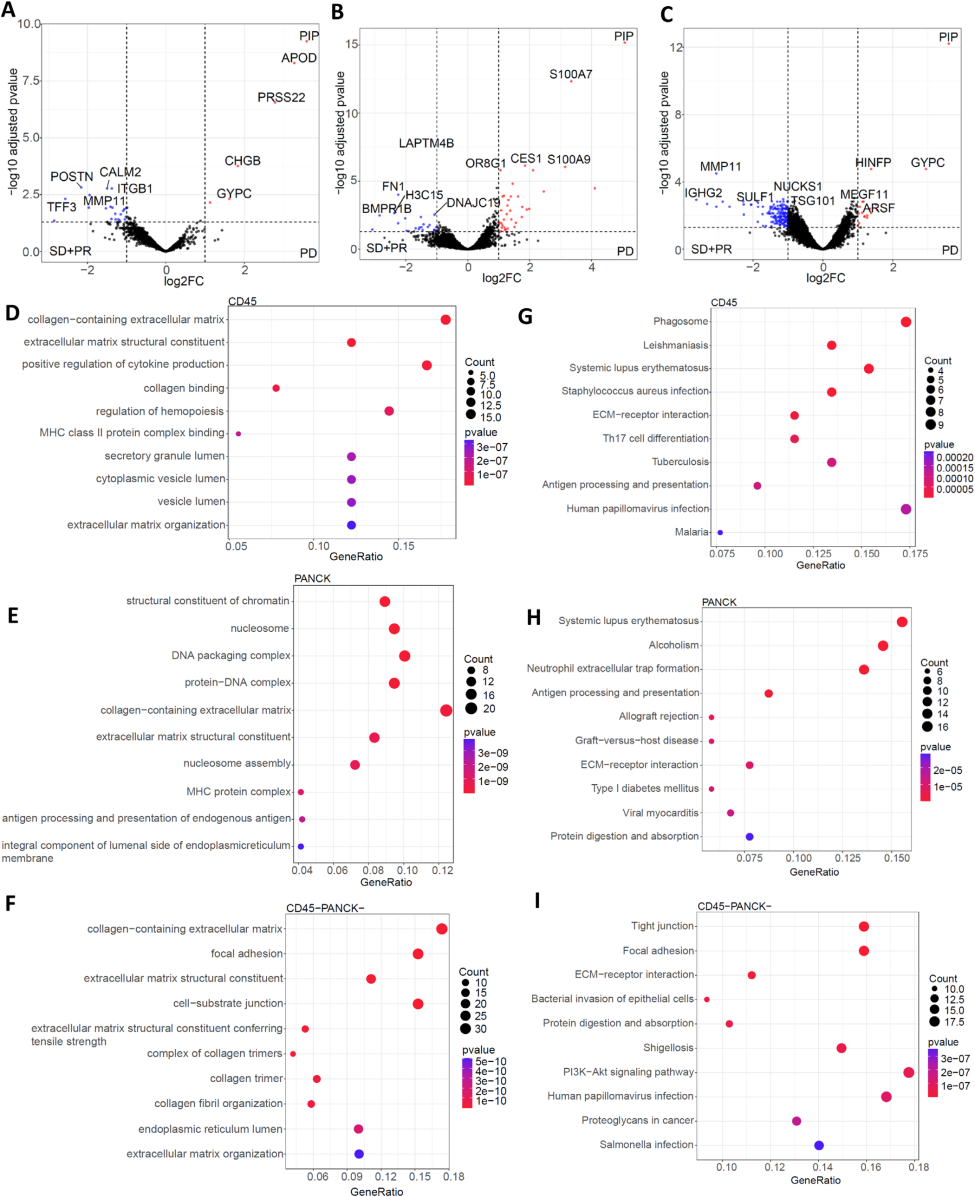
Volcano plots of DEGs and functional enrichment analysis. (A–C) Volcano plots showing the top five upregulated and top five downregulated DEGs between EVE‐sensitive and EVE‐resistant groups. The red dot represents the upregulated DEGs, the blue dot represents the downregulated DEGs, and the black dot represents genes without significance. (D–F) Top 10 GO enrichment annotations in spatially defined regions are summarized by bubble charts. The size of the bubble represents the number of DEGs enriched in biological process, cellular component, or molecular function. The color of the bubble represents enrichment significance. (G–I) KEGG pathway enrichment analysis of DEGs in spatially defined regions. The x‐axis illustrates enrichment factors, and the y‐axis illustrates the pathway terms.

Using the DSP Whole Transcriptome Atlas (WTA) panel, we analyzed the expression of over 18,000 genes in the CD45+, PANCK+, and CD45−/PANCK− zones. Differentially expressed genes (DEGs) were identified using a screening criterion of adjusted *p*‐value < 0.05 and |log2FC| > 1. We identified 93 DEGs in the CD45+ zones, 174 in the PANCK+ zones, and 197 in the CD45−/PANCK− zones (Figure 5). The top five DEGs ranked by adjusted *p*‐value are shown in Figures 6A–C. Notably, the prolactin‐induced protein (PIP) gene was consistently the most significantly upregulated gene in the CD45+, PANCK+, and CD45−/PANCK− zones of the EVE‐sensitive group compared to the EVE‐resistant group.

To explore the biological relevance of these gene sets, we performed gene ontology (GO) and Kyoto Encyclopedia of Genes and Genomes (KEGG) enrichment analyses for each spatial compartment. GO annotations were divided into biological process (BP), cellular component (CC), and molecular function (MF). The top 10 GO annotations are shown in Figure 6D–F, and KEGG pathway enrichments in Figure 6G–I.

In the CD45+ zone, 497 GO terms and 34 KEGG pathways were enriched. The dominant pathways involved cytokine production, MHC class II regulation, and extracellular matrix (ECM) interaction. KEGG analysis revealed significant enrichment in Th17 cell differentiation (*p* < 0.0001), antigen processing and presentation (*p* < 0.0001), and ECM‐receptor interaction (*p* < 0.0001).

In the PANCK+ zones, 352 GO terms and 24 KEGG pathways were enriched. Major BPs included MHC protein complex and ECM organization. KEGG pathways were primarily enriched in neutrophil extracellular trap formation (*p* < 0.0001), antigen processing and presentation (*p* < 0.0001), and ECM‐receptor interaction (*p* < 0.0001).

In the CD45−/PANCK− zones, 546 GO terms and 49 KEGG pathways were significantly enriched. Processes involving the ECM were highly enriched, with collagen‐containing ECM showing tremendous significance (*p* < 0.0001). Pathways such as tight junction (*p* < 0.0001), focal adhesion (*p* < 0.0001), ECM‐receptor interaction (*p* < 0.0001), and protein digestion and absorption (*p* < 0.0001) were significantly enriched. Importantly, the phosphatidylinositol 3‐kinase (PI3K)/Akt signaling pathway had the highest gene ratio, with 19 DEGs enriched.

### 2.4 High Expression of PIP mRNA Is Potentially Associated With Poorer Treatment Response to EVE

**FIGURE 7:**
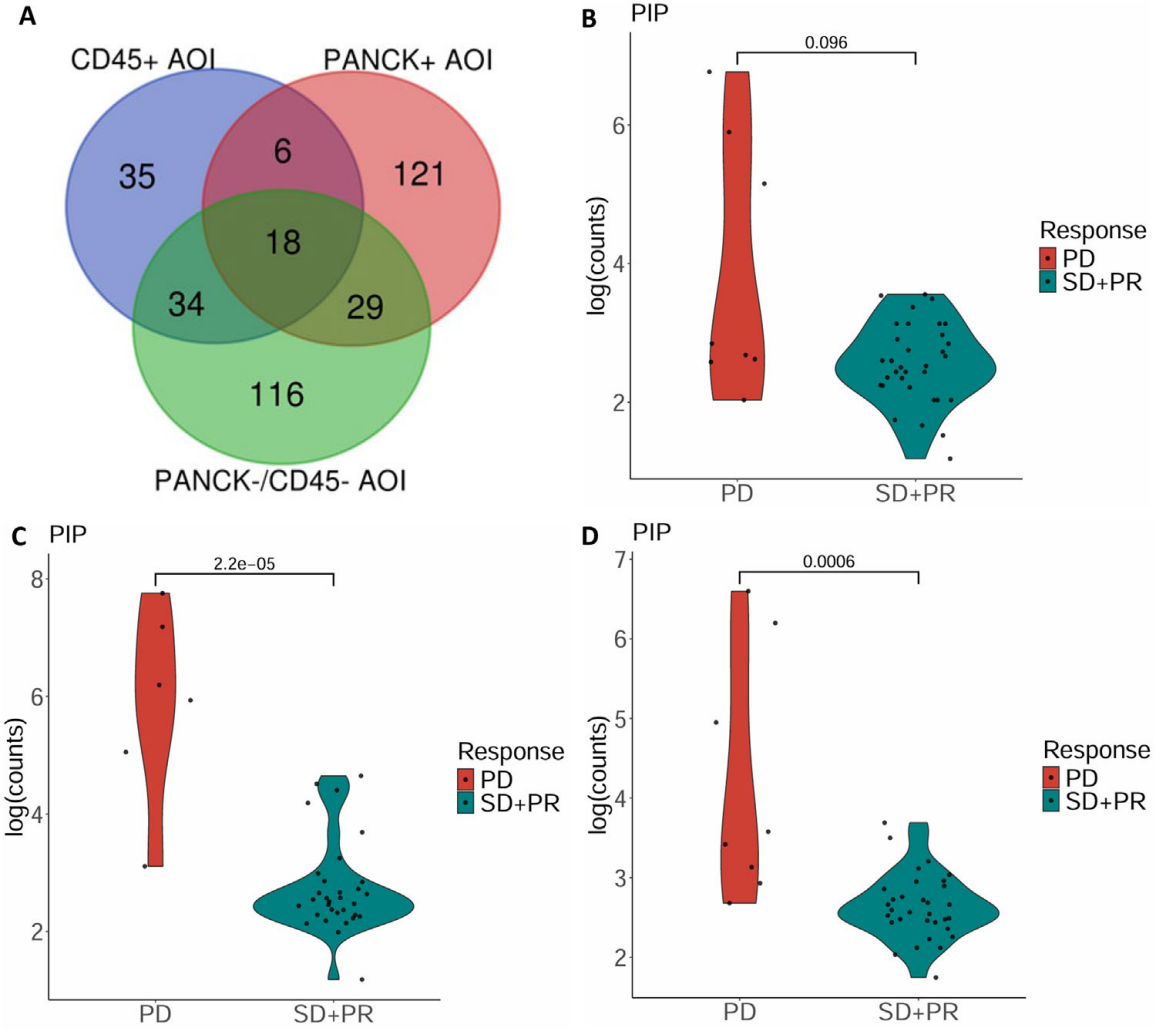
Among the DEGs, PIP emerged as the most significant. (A) Venn diagram showing 18 common DEGs among the CD45+ AOI, the PANCK+ AOI, and the CD45−/PANCK− AOI. AOI: area of interest. (B–D) Violin plots of PIP mRNA expression in patients who responded to EVE (SD+PR) versus those with PD. (B) CD45+ zone, (C) PANCK+ zone, and (D) CD45−/PANCK− zone.

We identified multiple DEGs between EVE‐sensitive and EVE‐resistant groups in the PANCK+, CD45+, and CD45−/PANCK− zones. The intersection of DEGs from all three zones resulted in 18 common genes (Figure 7A, Table S2). Among these genes, PIP showed the greatest differential expression and significance across all compartments. Specifically, it was highly upregulated in the EVE‐resistant group within the PANCK+ (log2FC = 5.1, *p* < 0.0001), CD45+ (log2FC = 3.6, *p* < 0.0001), and CD45−/PANCK− (log2FC = 3.6, *p* < 0.0001) zones (Table S2).

To further validate this observation, the Wilcoxon rank‐sum test was used to compare the mRNA expression of common DEGs between the EVE‐sensitive and EVE‐resistant groups (Figure S3). PIP expression was significantly elevated in the EVE‐resistant group in both the PANCK+ (*p* < 0.0001, Figure 7C) and CD45−/PANCK− (*p* = 0.0006, Figure 7D) zones. In the CD45+ zone, a trend toward higher expression was observed but did not reach statistical significance (*p* = 0.096, Figure 7B). These results reinforce the association between high PIP expression and poor clinical response.

To explore potential functional roles of PIP, we conducted GO and KEGG pathway analyses. In the BP category, PIP was associated with the negative regulation of apoptotic across all three zones. In the MF category, PIP was involved in actin binding, IgG binding, immunoglobulin binding, and aspartyl protease activity. No significant associations were found in the CC category or KEGG pathways. Details are provided in Table S3.

### 2.5 Association Between PIP mRNA Expression and Treatment Response or Survival Outcomes in HR+/HER2− ABC Patients Receiving EVE‐Combined Therapy

To explore the association between PIP mRNA expression and treatment response, logistic regression analyses were conducted. In the CD45+ zone, no significant correlation was observed (*p* = 0.235). In the PANCK+ zone and CD45−/PANCK− zone, univariate analyses revealed that elevated PIP expression might be linked to EVE resistance (OR = 1.009, 95% confidence interval [CI] = 1.000–1.018, *p* = 0.044; OR = 1.269, 95% CI = 1.051–1.532, *p* = 0.013). Nevertheless, neither association remained significant after multivariable adjustment. Additionally, age and metastatic site were identified as potential factors associated with resistance (Tables S4–S6).

**FIGURE 8:**
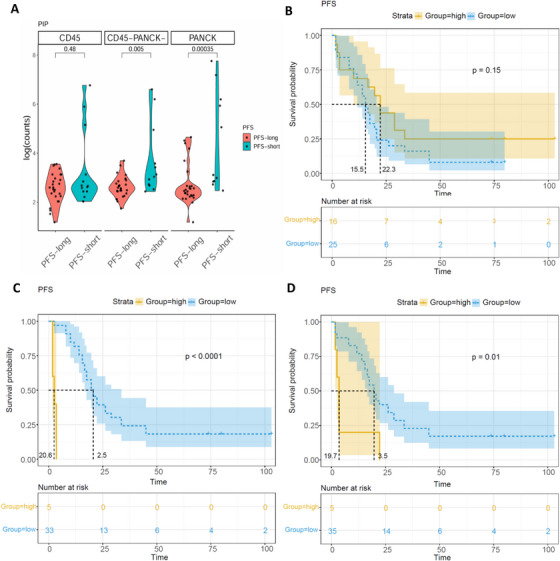
Relationship between PIP mRNA expression and PFS in patients. (A) Violin plots of PIP mRNA expression in spatially defined regions from patients with long (PFS ≥ 12 months) versus short (PFS < 12 months) PFS. ROI distribution was as follows: CD45+ zones, PFS ≥ 12 months (*n* = 27) and PFS < 12 months (*n* = 14); PANCK+ zones, PFS ≥ 12 months (*n* = 27) and PFS < 12 months (*n* = 11); CD45−/PANCK− zones, PFS ≥ 12 months (*n* = 28) and PFS < 12 months (*n* = 12). (B–D) Kaplan–Meier curves of PFS for patients with high PIP expression and low PIP expression. The function of surv_cutpoint in the surminer package was used to identify the optimal cut‐off value for determining PIP high expression and PIP low expression. (B) CD45+ zone, (C) PANCK+ zone, and (D) CD45−/PANCK− zone.

To evaluate the prognostic significance of PIP mRNA expression, we compared spatial expression levels in patients with short (<12 months) versus long (≥12 months) PFS. PIP mRNA expression in both the PANCK+ zone (*p* = 0.00035) and the CD45−/PANCK− zone (*p* = 0.005) was significantly higher in patients with short PFS. No significant difference was observed in the CD45+ zones (*p* = 0.48) (Figure 8A).

Subsequently, we conducted Kaplan–Meier (KM) survival analyses using PFS and overall survival (OS) as prognostic indicators. The optimal expression cutoffs for PFS and OS were determined using the surv_cutpoint function [20, 21], with thresholds of 15.27 and 22.90, respectively. At data cutoff, the median follow‐up was 79.8 months for both PFS and OS. A total of 17 patients experienced disease progression, and the median PFS was 17.5 months (95% CI = 11.3–23.8 months).

In the CD45+ zone, a trend toward worse PFS was observed in patients with high PIP expression (15.5 months vs. 22.3 months), but the difference was not statistically significant (*p* = 0.15, HR = 1.66, 95% CI = 0.82–3.37; Figure 8B). In the PANCK+ zone, patients with high PIP expression had a significantly shorter median PFS (2.5 months) compared to those with low expression (20.6 months; *p* < 0.0001, HR = 0.015, 95% CI = 0.0016–0.13; Figure 8C). Similarly, in the CD45−/PANCK− zone, high PIP expression was associated with shorter PFS (3.5 months vs. 19.7 months; *p* = 0.01, HR = 0.27, 95% CI = 0.099–0.72; Figure 8D). Regarding OS, 14 patients had died at the time of analysis, while the remaining seven patients were still receiving treatment for disease control. The median OS was 44.6 months (95% CI = 22.5–66.6 months). No significant association was observed between PIP mRNA expression and OS across the spatially resolved regions (Figure S4).

Furthermore, Cox regression analyses were conducted to evaluate the association between PIP expression and PFS. In the CD45+ and CD45−/PANCK− zones, univariate Cox models indicated significant associations (HR = 1.006, 95% CI = 1.000–1.012, *p* = 0.038; HR = 1.004, 95% CI = 1.001–1.007, *p* = 0.019). However, these associations did not remain significant in multivariable analyses. In the PANCK+ zone, no significant relationship was observed between PIP expression and PFS (Tables S7–S9).

Overall, these findings indicate that high PIP mRNA expression in tumor and stromal regions is strongly associated with poor PFS in HR+/HER2− ABC patients treated with EVE, but not with OS.

## 3 Discussion

Several studies have attempted to identify biomarkers of EVE efficacy in breast cancer through tissue or blood analyses. For instance, Hortobagyi et al. conducted correlative analyses of genetic alterations and EVE benefits using tumor specimens from a large subset of patients in the BOLERO‐2 study [12]. Nevertheless, no practical biomarkers were identified, consistent with other studies [13, 14]. One possible explanation for these results could be the limited attention paid to regulatory elements of the TME, which may modulate tumor behavior during EVE treatment. Our study aimed to overcome these limitations by utilizing high‐plex DSP technology. We explored potential biomarkers in three tissue compartments, offering a more integrated view of the interplay between oncogenic signaling and the immune microenvironment.

To our knowledge, this is the first study to investigate the association between EVE efficacy and spatial TME characteristics in patients with HR+/HER2− ABC. Our findings reveal distinct immune landscapes between EVE‐sensitive and EVE‐resistant groups. Through DSP, we identified PIP as a predictive biomarker of treatment response and survival outcomes in patients receiving EVE combined with letrozole.

The PIP gene encodes a 146‐amino acid glycoprotein (17 kDa) with aspartyl protease activity [22, 23]. PIP binds to multiple molecules—including IgG, actin, zinc α2‐glycoprotein (ZAG), and fibronectin—enabling it to perform different roles in disease processes. The role of PIP in breast cancer remains controversial. Several studies have reported tumor‐suppressive properties of PIP, including inhibition of malignant evolution, enhanced chemosensitivity, and improved survival outcomes [24, 25, 26, 27, 28]. In a mouse model of triple‐negative breast cancer (TNBC), overexpression of PIP in 4T1 cells led to reduced tumor growth and weight [25]. And in clinical cohorts, high PIP mRNA expression has been associated with improved disease‐free and metastasis‐free survival [27, 28]. However, other studies have indicated that PIP may promote cancer progression. For instance, the same 4T1 TNBC model with high PIP expression developed more metastatic lung colonies [25]. Additionally, in vitro drug sensitivity assays indicated that PIP was required for the proliferation and invasion of HR+ breast cancer cells resistant to tamoxifen treatment [26].

This apparent duality in PIP function may reflect its context‐dependent behavior across different breast cancer subtypes and treatment settings. In treatment‐naive or early‐stage tumors, PIP may act as a marker of differentiation or favorable biology. In contrast, under selective pressure from endocrine therapies or CDK4/6 inhibitors, PIP may acquire adaptive functions that support proliferation, or resistance. All patients in our MIRACLE trial had developed resistance to prior tamoxifen therapy before receiving EVE plus letrozole. Within this endocrine‐resistant context, high PIP expression was consistently associated with inferior treatment response and survival outcomes. These findings suggest that PIP may play a distinct role in advanced disease, potentially contributing to resistance mechanisms specific to mTOR inhibition or altered hormonal signaling. Further research is needed to elucidate the molecular mechanisms underlying this functional duality.

To explore explanations for these observations, we propose several hypotheses based on prior literature and our pathway analyses. First, PIP may indirectly enhance the activity of the PI3K/Akt/mTOR (PAM) signaling pathway through the estrogen receptor (ER) pathway. Prior studies have shown that PIP is highly expressed in HR+/HER2− breast cancer and correlates positively with ER and PR expression [26, 27, 28, 29]. Since EVE can reverse endocrine resistance via inhibition of ER signaling, high PIP expression may partially undermine this effect by maintaining ER activity, thereby promoting resistance to mTOR inhibition [30, 31]. Second, PIP may promote crosstalk between prolactin receptor signaling and the PAM pathway, thereby contributing to EVE resistance. Prolactin has been shown to activate the PI3K/Akt/mTOR pathway in a dose‐ and time‐dependent manner in lymphoma models. Given that PIP may enhance prolactin signaling, this could contribute to sustained mTOR activation even in the presence of EVE [32, 33].

While our data suggest a potential association between PIP expression and resistance to EVE, we acknowledge that the mechanism remains speculative. We did not conduct functional experiments such as PIP knockdown or overexpression. Therefore, any role of PIP in endocrine or mTOR inhibitor resistance should be interpreted as a hypothesis and warrants further experimental validation. From a translational standpoint, clinical implementation of PIP mRNA as a predictive biomarker will require rigorous validation and standardized detection methods. Although DSP provides high‐resolution spatial data, its cost and complexity limit routine clinical use. Once validated, PIP expression could be assessed using more accessible methods such as qRT‐PCR, RNA‐ISH, or immunohistochemistry (IHC) on FFPE biopsy samples. Clinically, PIP may complement existing biomarkers like PIK3CA mutations, which guide PI3K inhibitor use but do not predict EVE response.

Meanwhile, accumulating evidence suggests that remodeling of the TME may influence the efficacy of EVE. Fibroblasts, particularly cancer‐associated fibroblasts (CAFs), have multiple roles in modulating drug delivery, ECM organization, and immune regulation [34]. CAFs have traditionally been regarded as key promoters of tumor progression. However, recent studies have shown that certain CAF subtypes can exert mechanical effects that suppress tumor dissemination [35, 36]. In our study, tumors with more fibroblasts had better response to EVE; this suggests a supportive stromal structure for mTOR inhibitors. In contrast, elevated neutrophil infiltration in EVE‐resistant tumors may reflect a more immunosuppressive or pro‐inflammatory TME. Neutrophils can secrete cytokines, promote angiogenesis, suppress T‐cell activity, and induce epithelial–mesenchymal transition (EMT)—all of which have been implicated in resistance to targeted therapies [37, 38]. Their presence may also indicate systemic inflammation or metabolic reprogramming unfavorable to mTOR inhibition efficacy. Moreover, increased plasma cells observed in resistant cases may represent an imbalanced immune state that promotes tolerance rather than cytotoxicity [39]. Overall, our findings suggest that the immune microenvironment may contribute to treatment heterogeneity.

### 3.1 Limitations and Future Directions

This study has some limitations. First, our cohort's small sample size may influence the results and limit the ability to detect subtle differences. Spatial transcriptomic studies employing DSP technology often face sample size constraints due to tissue availability and technical complexity. Nevertheless, prior research, such as Song et al. [40], has successfully utilized small DSP cohorts for biomarker discovery [40]. Second, the study lacked a validation cohort to confirm PIP's predictive role in EVE's clinical efficacy. To facilitate external validation of PIP expression, we searched for public datasets. While no dataset exactly matches the treatment context of EVE plus letrozole in HR+/HER2− ABC, one related dataset is available. Specifically, pre‐ and post‐treatment expression profiles from patients receiving EVE (GSE119262) can serve as a valuable resource. Given the exploratory nature of this study, our primary focus was on hypothesis generation. In the future, we aim to leverage this dataset to further explore the dynamic expression changes of PIP before and after EVE‐based treatment, which may provide deeper insight into the functional role of PIP in mediating treatment response. In addition, retrospective analyses of existing clinical trial samples (e.g., from BOLERO‐2) and prospective clinical trials incorporating biomarker stratification could be conducted. Third, we used TMA for spatial quantification rather than whole‐tissue sections. While we investigated two nonadjacent tumor cores per patient, we acknowledge that this limited sampling may not represent the entire tumor. TME heterogeneity may influence the performance of biomarkers, particularly those with heterogeneous immune expression. Therefore, future research should involve whole‐tissue section analyses with more extensive sampling. Last, in the regression models, the limited sample size and potential multicollinearity led to unstable convergence in some multivariable models, with complete or near‐complete separation observed. Therefore, the interpretation primarily relied on univariate results, which should be considered with caution. At present, PIP should be regarded as a correlational rather than an independent predictive biomarker.

## 4 Conclusion

In conclusion, our study highlights the distinct spatial immune landscapes between HR+/HER2− ABC patients exhibiting different responses to EVE, and supports the use of transcriptomic biomarkers to stratify patients likely to benefit from EVE combined with letrozole.

## 5 Materials and Methods

### 5.1 MIRACLE Trial and Patients

The multicenter phase 2 MIRACLE trial enrolled 199 premenopausal patients with HR+/HER2− ABC, who were randomly assigned in a ratio of 1:1 to receive EVE plus letrozole (101 patients) or letrozole alone (98 patients). All patients experienced disease recurrence within 24 months after completion of adjuvant tamoxifen treatment or disease progression while receiving advanced tamoxifen treatment. The study has reached its primary endpoint of PFS. It showed that patients had a significantly improved PFS of 19.4 months when receiving EVE plus letrozole. The results were reported separately [11].

The clinicopathological characteristics of patients in the EVE arm were retrospectively collected from medical records. Based on Response Evaluation Criteria in Solid Tumors (RECIST) 1.1, treatment response was evaluated as complete response (CR), PR, SD, and PD. The optimal response to EVE serves as the basis for grouping. Patients developing PD or SD for less than 6 months are defined as the EVE‐resistant group, while patients achieving CR or PR or SD for more than 6 months were defined as the EVE‐sensitive group.

### 5.2 Samples and TMA

Postoperative FFPE tumor samples of patients in the EVE arm were collected from the tissue bank of the Department of Pathology, Cancer Hospital Chinese Academy of Medical Sciences. All patients’ HE slides were reviewed by two independent expert pathologists (X and W). Two tumor cores from archived FFPE tissues of each patient were circled and punched. Each tumor core complied with the testing requirements, with a max diameter of 1 mm. TMA of two tumor cores per patient was then constructed. It was approximately arranged in a five‐column, nine‐row format.

### 5.3 Slide Preparation

The TMA block was continuously sliced with a thickness of less than 5um and mounted on the slip‐resistant SuperFrostPlus glass slides (Thermo scientific 12‐550‐15). The first few pieces of sliced sections were discarded. Tissue sections were placed in the Scan Area in the center of the slide to ensure complete coverage during the imaging process. Generally, the TMA sections with tissue covering areas of a length less than 36.2 mm and a width not exceeding 14.6 mm were baked in a drying oven in a vertical format, allowing complete draining of paraffin. This process was conducted at 60°C for 60 min before deparaffinization.

### 5.4 Sample Processing

For DSP, TMA mounted on slides was preprocessed in a stepwise procedure involving deparaffinization, rehydration and antigen retrieval. Generally, tissues were dewaxed in limonene for 5 min, repeated twice with replacement of the dewaxing reagent, and subsequently rehydrated in gradient ethanol (100%, 95%, and 70%, respectively) before antigen retrieval. The retrieval process was undertaken in a designated pressure cooker (BioSB, USA) using Tris EDTA buffer (pH = 9.0, Thermo Scientific, USA) at 100°C for 15 min without pressure induction according to manufacturer's recommendation. Upon this, RNA targets were exposed using protease K (Thermo Scientific, USA) at 1 µg/mL concentration for 15 min to allow removal of high‐ordered protein structures bound to RNA. Tissues were then fixed in 10% neutral buffered formaldehyde (Thermo Fisher Scientific, USA) for 5 min and terminated in NBF‐stopping buffer.

### 5.5 In Situ Hybridization Using WTA Panel

Processed TMA slides were incubated at 37°C overnight with a mixture of pre‐designed oligo using the DSP‐WTA (GeoMx Whole Transcriptome Atlas, NanoString, USA) in a hybridization chamber (Boekel Scientific RapidFISH, USA). Excessive probes were removed. The GeoMx Morphology Kit for RNA (NanoString, USA) was used for tissue visualization to guide the selection of ROI. The antibodies used as morphology markers were Pan‐cytokeratin (PANCK, Novus NEP2–33200) for tumor and epithelial cells, CD45 (CST13917) for leucocytes, plus a nuclear staining SYTO13 (NanoString 121300310).

### 5.6 Choosing ROI

The resulting tissue slides were loaded onto a DSP instrument and scanned to obtain high‐quality multi‐selection. The slides were picked with essential criteria of 38,000 and 380,000 µm^2^ size with a minimal estimate of 100 cells roughly as analytical inputs. Tissue‐bound target‐specific oligos were released via photocleavable linkers where UV lights were illuminated. The oligo probes were subsequently collected into a 96‐well collection plate via a capillary sipper. Slides were re‐mounted by the Fluoromount G mounting medium for other pathological inspection purposes (Northern Biotech, Waltham, USA). ROIs, including tumor, stromal, and immune‐infiltrated areas, were defined by two experienced pathologists based on fluorescence antibody staining (PANCK and CD45) from preliminary experiments.

### 5.7 DSP‐WTA Readouts Generated via NGS

In brief, to generate an NGS‐compatible sequencing library, the collected probes were hybridized with barcode‐indexed primer sets and the reactions were conducted with a Master Mix containing the necessary components (including enzymes and dNTPs) for library construction. The purified sequencing library was obtained according to standard protocols. Quality control was checked with QFX (Denovix, USA) and Qsep100 (BIOPTIC, CHINA) for fragment length, which peaks at around 172 bp. NGS was performed on an illuminated Nova‐seq 6000 system.

### 5.8 Data Quality Control and Normalization

Raw sequencing data from different ROIs were obtained and processed on the DSP analysis system with a demultiplexing process to assign raw sequencing reads (FASTQ) into designated ROIs. Data QC of ROIs and targets was conducted to filter unsatisfied ROIs based on suggested QC criteria. The QC involved several technical measures, including signal QC, technical background QC, DSP parameters, and low‐expression probe QC. The target QC was used to identify gene expression based on the limit of quality (LOQ). The equation for LOQ was listed as follows and the threshold was set as 2.0.

### 5.9 LOQ = GeoMean(NegProbe) × GeoSD(NegProbe)^threshold^

The raw counts were normalized using the Q3 normalization method, with the gene expression level at the 75th percentile as the reference.

### 5.10 Bioinformatics Analysis

The Deseq2 method was used to compare mRNA expression differences between groups to identify DEGs. The SpatialDecon tool developed for DSP transcriptomic data was used to quantify the cell populations in the TME based on normalized gene expression data from each segment per ROI. The SpatialDecon tool applied the constrained log‐normal regression algorithm, which was more in line with the long‐tailed characteristics of gene expression data. The GO annotation analysis and KEGG pathway analysis were performed using the clusterProfiler package of R software.

### 5.11 Statistical Analysis

The differences in clinicopathological characteristics (categorical variables) between patients in the EVE‐resistant and EVE‐sensitive groups were compared using the chi‐squared or Fisher's exact test. The Wilcox rank‐sum test was used to generate box plots of the target DEGs between the two groups. The association between mRNA expression of the target DEGs and survival outcomes (including PFS and OS) was analyzed using the KM method. To determine the optimal cut‐off value for dividing patients into high and low PIP mRNA expression groups, we used the “surv_cutpoint()” function from the R package “survminer.” Logistic and Cox regression analyses were used to examine the association of PIP with response to EVE and patient prognosis. Hazard ratio (HR) and 95% CI were calculated via the log‐rank test. A *p*‐value less than 0.05 was considered statistically significant, and a *p*‐value less than 0.1 was considered to indicate a tendency toward significance, both of which regarded as false discovery rate (FDR) under appropriate contexts. SPSS (version 22.0) and R (version 4.2.0) software were used for all statistical analysis.

## Author Contributions

Ying Fan, Liyan Xue, and Binghe Xu conceptualized and designed the study. Jiayu Wang, Fei Ma, Yang Luo, Bo Lan, Pin Zhang, and Jianming Ying coordinated clinical sample collection and patient data acquisition. Yuhang Han, Danyang Ji, and Yujing Tan performed spatial transcriptomic data processing and statistical analyses. Yuhang Han, Danyang Ji, and Yujing Tan drafted the manuscript. All authors critically reviewed and approved the final version. All listed authors have contributed to the manuscript and have agreed to the final submitted version.

## Funding

This study was supported by the CAMS Innovation Fund for Medical Sciences (CIFMS) 2021‐12M‐1‐014 & 2023‐I2M‐C&T‐B‐077, the Major project of Medical Oncology Key Foundation of Cancer Hospital Chinese Academy of Medical Sciences (CICAMS‐MOMP202203), the Beijing Natural Science Foundation (L258033) and the National High Level Hospital Clinical Research Funding (2025‐LYZX‐D‐A02).

## Conflicts of Interest

The authors declare no conflicts of interest.

## Ethics Statement

This study was approved by the ethics committee of the National Cancer Center/National Clinical Research Center for Cancer/Cancer Hospital, Chinese Academy of Medical Sciences & Peking Union Medical College. All patients provided written informed consent prior to participation in the MIRACLE trial (ClinicalTrials.gov Identifier: NCT02313051).

## Supporting information




**Figure S1** Images showing ROIs in the tissue microarray for DSP analyses. (Left) Haematoxylin and eosin staining for preliminary screening. (Right) Compartmentalized images created by fluorescence in situ hybridization. Color of blue, green, and rose red represents the CD45+ zone, the PANCK+ tumor zone, and the CD45−/PANCK− zone, respectively. The area within the circle or the irregular shape represents one ROI. Each row contains two ROIs from the same patient, arranged in order from left to right. ROI‐039 and ROI‐040 were selected from one tumor core of a patient in the EVE‐sensitive group since another tumor core was floated and was not qualified for hematoxylin and eosin staining or fluorescence colocalization.
**Figure S2** Scatter diagram showing the number of targets (genes) above and below the limit of quantitation (LOQ) per ROI for the DSP RNA assay. The equation for LOQ is presented as follows, *LOQ = GeoMean(NegProbe)×GeoSD(NegProbe)* ^2^. **A** CD45+ zone, **B** PANCK+ zone, **C** CD45−/PANCK− zone.
**Figure S3** Violin plots showing mRNA expression of 18 common DEGs in patients who responded to EVE (SD+PR) versus those with PD. **A** CD45+ zone, **B** PANCK+ zone, **C** CD45−/PANCK− zone.
**Figure S4** Kaplan–Meier curves showing OS for patients with high PIP expression versus low PIP expression. OS overall survival. The function of surv_cutpoint in the surminer package was used to identify the optimal cut‐off value for determining PIP high expression and PIP low expression. **A** CD45+ zone, **B** PANCK+ zone, **C** CD45−/PANCK− zone.
**Table S1** Counts of spatially defined zones for digital spatial profiling analysis
**Table S2** Eighteen common genes in spatially defined zones calculated by the Deseq2 method
**Table S3** GO and KEGG pathway annotation analysis on the crucial gene of PIP
**Table S4** Univariate and multivariate logistic regression in CD45+ regions
**Table S5** Univariate and multivariate logistic regression in PANCK+ regions
**Table S6** Univariate and multivariate logistic regression in CD45−/PANCK− regions
**Table S7** Univariate and multivariate Cox regression in CD45+ regions
**Table S8** Univariate and multivariate Cox regression in PANCK+ regions
**Table S9** Univariate and multivariate Cox regression in CD45−/PANCK− regions

## Data Availability

Raw data from the DSP analysis have been uploaded to the China National Center for Bioinformation—National Genomics Data Center (CNCB‐NGDC).
