# Supplementary material for: High‐Plex Digital Spatial Profiling Identified Prolactin‐Induced Protein mRNA Associated With Response and Survival of Everolimus and Letrozole Treatment for Hormone Receptor‐Positive/Human Epidermal Growth Factor Receptor 2‐Negative Advanced Breast Cancer
Source: MedComm (2020). 2025 Nov 26;6(12):e70509. doi: 10.1002/mco2.70509 (PMC12657622; doi:10.1002/mco2.70509)
Supplement: Supplementary file 1 — Figure S1 Images showing ROIs in the tissue microarray for DSP analyses. (Left) Haematoxylin and eosin staining for preliminary screening. (Right) Compartmentalized images created by fluorescence in situ hybridization. Color of blue, green, and rose red represents the CD45+ zone, the PANCK+ tumor zone, and the CD45−/PANCK− zone, respectively. The area within the circle or the irregular shape represents one ROI. Each row contains two ROIs from the same patient, arranged in order from left to right. ROI‐039 and ROI‐040 were selected from one tumor core of a patient in the EVE‐sensitive group since another tumor core was floated and was not qualified for hematoxylin and eosin staining or fluorescence colocalization. Figure S2 Scatter diagram showing the number of targets (genes) above and below the limit of quantitation (LOQ) per ROI for the DSP RNA assay. The equation for LOQ is presented as follows, LOQ = GeoMean(NegProbe)×GeoSD(NegProbe) 2. A CD45+ zone, B PANCK+ zone, C CD45−/PANCK− zone. Figure S3 Violin plots showing mRNA expression of 18 common DEGs in patients who responded to EVE (SD+PR) versus those with PD. A CD45+ zone, B PANCK+ zone, C CD45−/PANCK− zone. Figure S4 Kaplan–Meier curves showing OS for patients with high PIP expression versus low PIP expression. OS overall survival. The function of surv_cutpoint in the surminer package was used to identify the optimal cut‐off value for determining PIP high expression and PIP low expression. A CD45+ zone, B PANCK+ zone, C CD45−/PANCK− zone. Table S1 Counts of spatially defined zones for digital spatial profiling analysis Table S2 Eighteen common genes in spatially defined zones calculated by the Deseq2 method Table S3 GO and KEGG pathway annotation analysis on the crucial gene of PIP Table S4 Univariate and multivariate logistic regression in CD45+ regions Table S5 Univariate and multivariate logistic regression in PANCK+ regions Table S6 Univariate and multivariate logistic regression in CD45−/PANCK− regions T [file MCO2-6-e70509-s001.docx]

**High-plex digital spatial profiling identified PIP mRNA associated with response and survival of everolimus and** **letrozole treatment for hormone receptor-positive/human epidermal growth factor receptor 2-negative advanced** **breast cancer**

Yuhang Han^1#^, Danyang Ji^1#^, Yujing Tan^1#^, Jiayu Wang^1^, Fei Ma^1^, Yang Luo^1^, Bo Lan^1^, Pin Zhang^1^, Jianming Ying^2^, Binghe Xu^1*^, Liyan Xue^2*^, Ying Fan^1*^

**Supplementary Information**


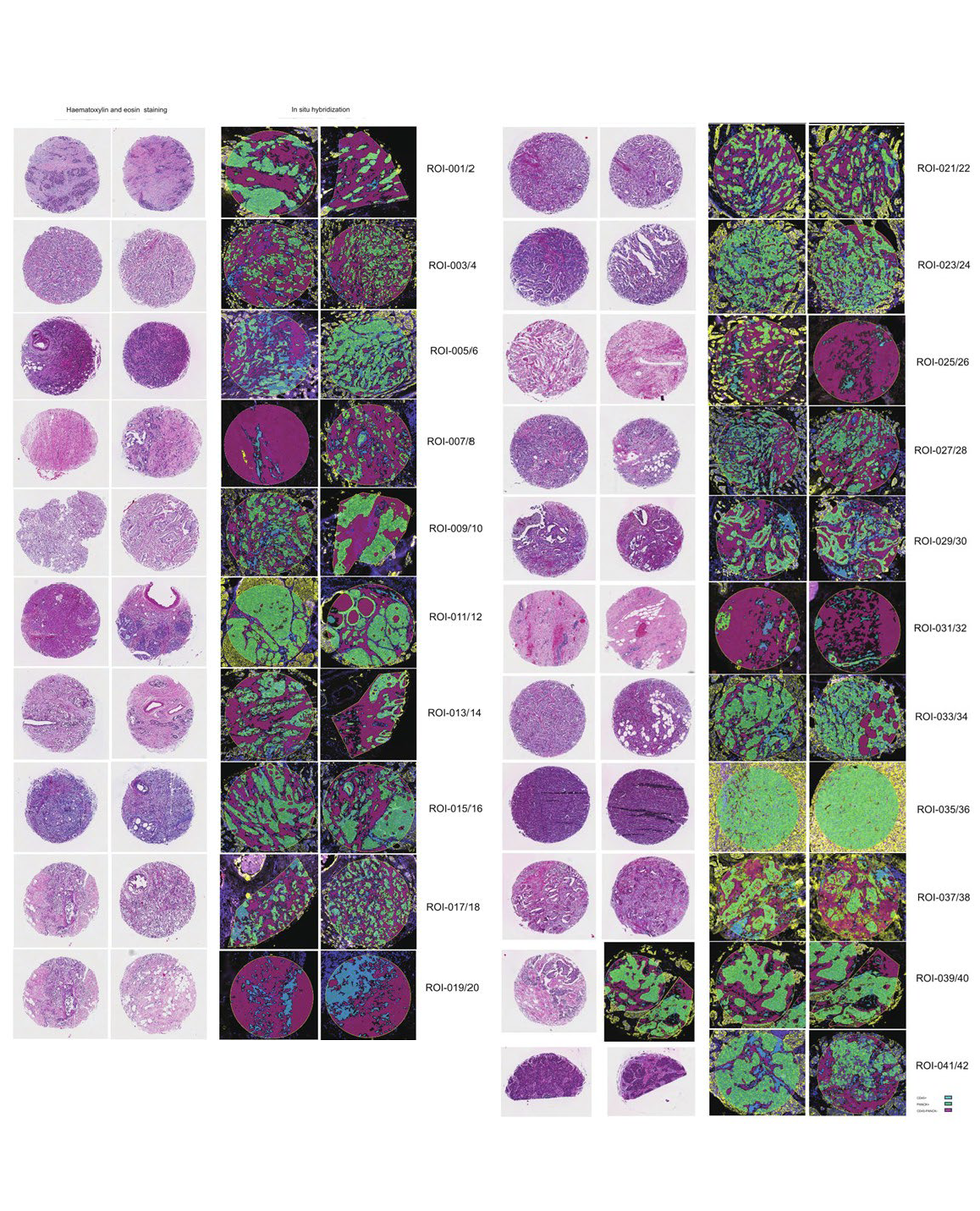
 **Figure S1** Images showing ROIs in the tissue microarray for DSP analyses. (Left) Haematoxylin and eosin staining for preliminary screening. (Right) Compartmentalized images created by fluorescence in situ hybridization. Color of blue, green, and rose red represents the CD45+ zone, the PANCK+ tumor zone, and the CD45−/PANCK− zone, respectively. The area within the circle or the irregular shape represents one ROI. Each row contains two ROIs from the same patient, arranged in order from left to right. ROI-039 and ROI-040 were selected from one tumor core of a patient in the EVE-sensitive group since another tumor core was floated and was not qualified for hematoxylin and eosin staining or ﬂuorescence colocalization.


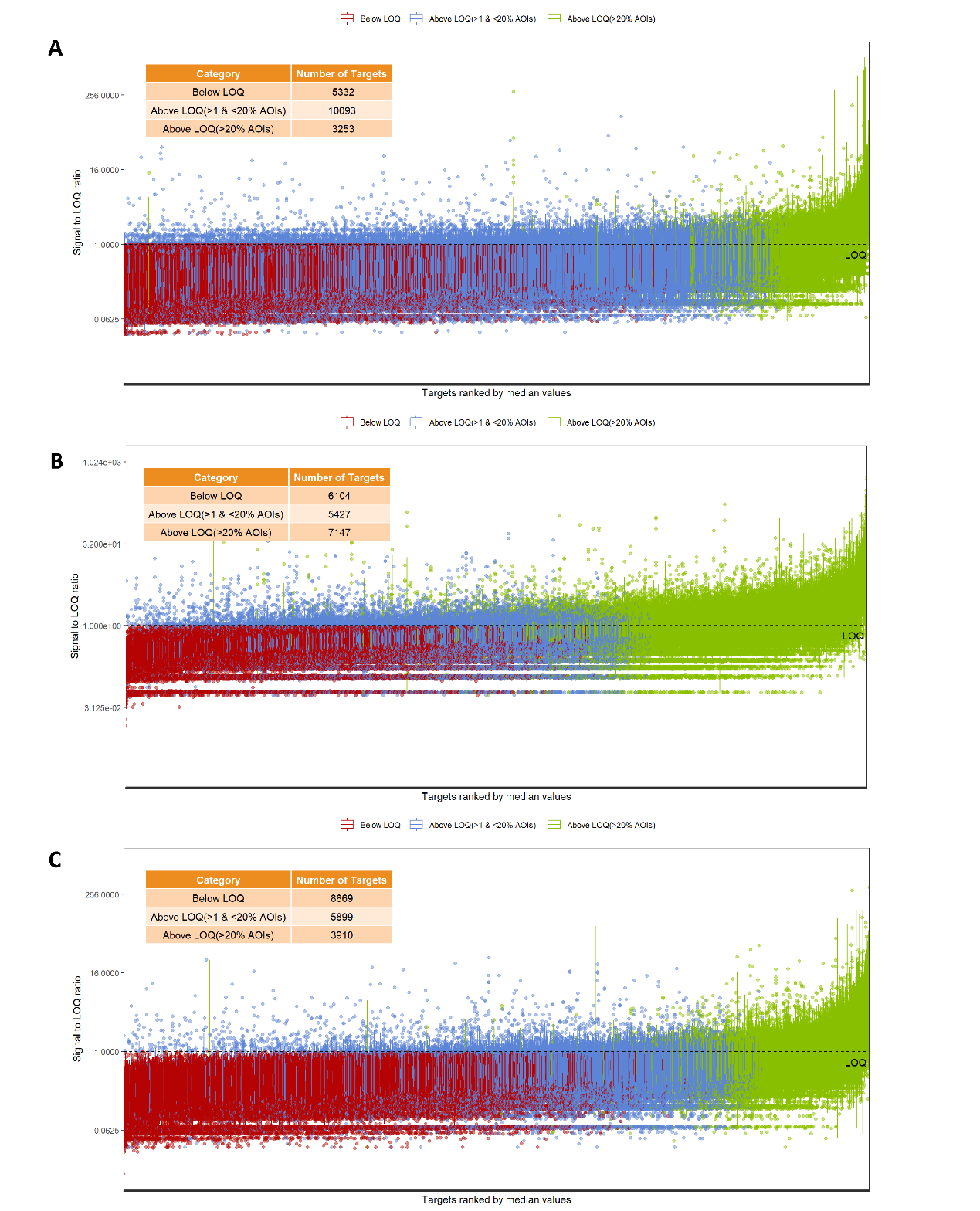
 **Figure S2** Scatter diagram showing the number of targets (genes) above and below the limit of quantitation (LOQ) per ROI for the DSP RNA assay. The equation for LOQ is presented as follows, *LOQ=GeoMean(NegProbe)×GeoSD(NegProbe)^2^*. **A** CD45+ zone, **B** PANCK+ zone, **C** CD45−/PANCK− zone.


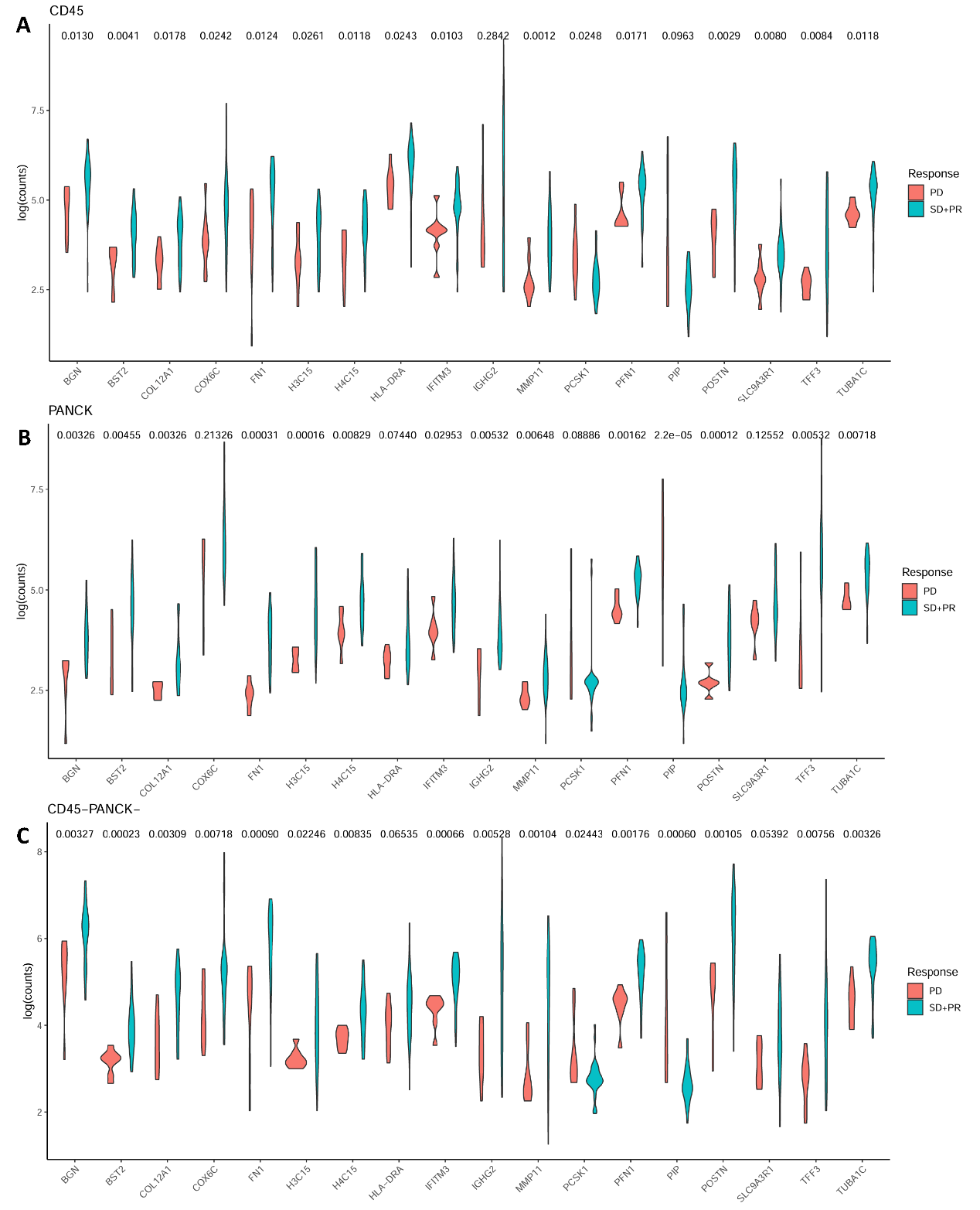
 **Figure S3** Violin plots showing mRNA expression of 18 common DEGs in patients who responded to EVE (SD+PR) versus those with PD. **A** CD45+ zone, **B** PANCK+ zone, **C** CD45−/PANCK− zone.


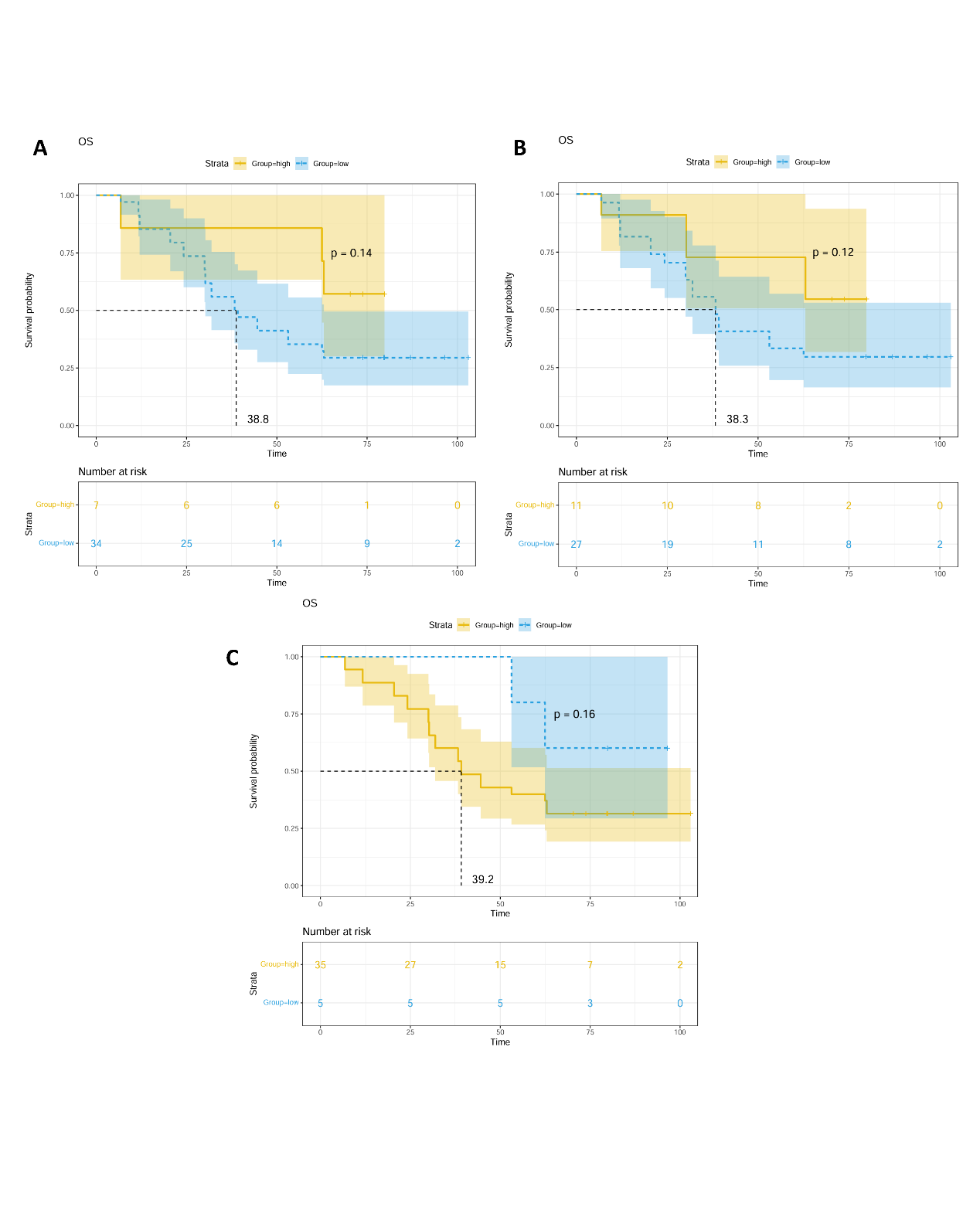
 **Figure S4** Kaplan–Meier curves showing OS for patients with high PIP expression versus low PIP expression. OS overall survival. The function of surv_cutpoint in the surminer package was used to identify the optimal cut-off value for determining PIP high expression and PIP low expression. **A** CD45+ zone, **B** PANCK+ zone, **C** CD45−/PANCK− zone.

**Table S1** Counts of spatially defined zones for digital spatial profiling analysis

| **Counts** | **CD45+ zone** | **PANCK+ zone** | **CD45-/PANCK- zone** |
| --- | --- | --- | --- |
| Total counts | 41 | 38 | 40 |
| EVE-sensitive counts | 33 | 32 | 32 |
| EVE-resistant counts | 8 | 6 | 8 |

**Table S2** Eighteen common genes in spatially defined zones calculated by the Deseq2 method

| **Gene symbol** | **CD45+ zone** | | **PANCK+ zone** | | **CD45-/PANCK- zone** | |
| --- | --- | --- | --- | --- | --- | --- |
|  | ***Log 2FC*** | ***P value*** | ***Log 2FC*** | ***P value*** | ***Log 2FC*** | ***P value*** |
| BGN | -1.18959 | 0.002202 | -1.65324 | 0.000149 | -1.34564 | 0.000294 |
| BST2 | -1.5138 | 7.88E-06 | -1.76806 | 0.001136 | -1.4063 | 3.79E-05 |
| COL12A1 | -1.28641 | 0.000336 | -1.4825 | 0.002216 | -1.5339 | 7.29E-05 |
| COX6C | -1.92952 | 0.000929 | -1.66994 | 0.007877 | -2.2583 | 0.000109 |
| FN1 | -1.47168 | 0.001191 | -2.23436 | 6.22E-08 | -2.04773 | 4.81E-06 |
| H3C15 | -1.18086 | 0.001724 | -2.36932 | 3.41E-06 | -1.73232 | 0.000107 |
| H4C15 | -1.1625 | 0.000316 | -1.23 | 0.001798 | -1.16204 | 0.000324 |
| HLA-DRA | -1.00623 | 0.006365 | -1.19202 | 0.011877 | -1.01118 | 0.010372 |
| IFITM3 | -1.17779 | 0.00067 | -1.16726 | 0.008065 | -1.12806 | 4.7E-05 |
| IGHG2 | -1.54881 | 0.042104 | -1.84495 | 0.000571 | -3.61877 | 1.98E-07 |
| MMP11 | -2.06185 | 7.03E-06 | -1.09565 | 0.00437 | -3.05481 | 1.82E-08 |
| PCSK1 | 1.186903 | 7.24E-05 | 1.912368 | 0.007692 | 1.169586 | 0.000154 |
| PFN1 | -1.04794 | 0.000898 | -1.0261 | 0.000211 | -1.24322 | 1.43E-05 |
| PIP | 3.583054 | 2.55E-12 | 5.061014 | 1.25E-20 | 3.581847 | 2.01E-18 |
| POSTN | -2.17864 | 1.83E-06 | -1.99487 | 1.46E-05 | -2.27039 | 1.33E-06 |
| SLC9A3R1 | -1.30635 | 0.002071 | -1.03396 | 0.02139 | -1.47026 | 0.001545 |
| TFF3 | -2.61377 | 3.06E-05 | -3.10818 | 0.000155 | -3.31941 | 1.21E-06 |
| TUBA1C | -1.0193 | 0.001023 | -1.01886 | 0.003235 | -1.29398 | 5.2E-05 |

Abbreviations: FC fold change.

**Table S3** GO and KEGG pathway annotation analysis on the crucial gene of PIP

| **Category** | **PANCK+ zone** | **CD45+ zone** | **CD45-/PANCK- zone** |
| --- | --- | --- | --- |
| GO-BP | detection of chemical stimulus involved in sensory perception | negative regulation of leukocyte apoptotic process | sensory perception of taste |
|  | detection of chemical stimulus involved in sensory perception of bitter taste | regulation of leukocyte apoptotic process | detection of chemical stimulus involved in sensory perception of bitter taste |
|  | detection of chemical stimulus involved in sensory perception of taste | leukocyte apoptotic process | negative regulation of T cell apoptotic process |
|  | negative regulation of leukocyte apoptotic process | lymphocyte apoptotic process | negative regulation of leukocyte apoptotic process |
|  | sensory perception of bitter taste | regulation of lymphocyte apoptotic process | negative regulation of lymphocyte apoptotic process |
|  | regulation of leukocyte apoptotic process | negative regulation of lymphocyte apoptotic process | sensory perception of bitter taste |
|  | sensory perception of taste | T cell apoptotic process | T cell apoptotic process |
|  | leukocyte apoptotic process | regulation of T cell apoptotic process | detection of chemical stimulus involved in sensory perception of taste |
|  | negative regulation of lymphocyte apoptotic process | sensory perception of bitter taste | regulation of T cell apoptotic process |
|  | regulation of lymphocyte apoptotic process | tissue homeostasis | regulation of lymphocyte apoptotic process |
|  | tissue homeostasis | retina homeostasis | lymphocyte apoptotic process |
|  | lymphocyte apoptotic process | detection of chemical stimulus involved in sensory perception of bitter taste | regulation of leukocyte apoptotic process |
|  | anatomical structure homeostasis | detection of chemical stimulus involved in sensory perception of taste | leukocyte apoptotic process |
|  | negative regulation of T cell apoptotic process | sensory perception of taste | tissue homeostasis |
|  | T cell apoptotic process | negative regulation of T cell apoptotic process | anatomical structure homeostasis |
|  | retina homeostasis | anatomical structure homeostasis | retina homeostasis |
|  | regulation of T cell apoptotic process | detection of chemical stimulus involved in sensory perception | detection of chemical stimulus involved in sensory perception |
| GO-MF | actin binding | actin binding | actin binding |
|  | aspartic-type endopeptidase activity | IgG binding | immunoglobulin binding |
|  | aspartic-type peptidase activity | immunoglobulin binding | endopeptidase activity |
|  | endopeptidase activity | endopeptidase activity | IgG binding |
|  | IgG binding | aspartic-type endopeptidase activity | aspartic-type endopeptidase activity |
|  | immunoglobulin binding | aspartic-type peptidase activity | aspartic-type peptidase activity |
| GO-CC | None | None | None |
| KEGG pathway | None | None | None |

Abbreviations: GO Gene Ontology, KEGG Kyoto Encyclopedia of Genes and Genomes, BP biological process, CC cellular component, MF molecular function.

| **Characteristics** | **Univariate analysis** | | | | **Multivariate analysis** | | | | |
| --- | --- | --- | --- | --- | --- | --- | --- | --- | --- |
|  | **OR** | **CI95% lower** | **CI95% upper** | **P value** | **OR** | **CI95% lower** | **CI95% upper** | **P value** |  |
| **Age** | 1.799 | 1.115 | 2.903 | 0.016* | 1.366 | 0.935 | 1.994 | 0.107 |  |
| **Histological grade** | 1.16E+17 | 0 | inf | 1 |  |  |  |  |  |
| **T stage** | 0.472 | 0.081 | 2.752 | 0.404 |  |  |  |  |  |
| **N stage** | 0.633 | 0.277 | 1.448 | 0.279 |  |  |  |  |  |
| **M stage** | 0 | 0 | inf | 1 |  |  |  |  |  |
| **ER status** | 0.625 | 0.097 | 4.047 | 0.622 |  |  |  |  |  |
| **PR status** | 0.935 | 0.434 | 2.016 | 0.865 |  |  |  |  |  |
| **HER2 status** | 0.548 | 0.182 | 1.646 | 0.284 |  |  |  |  |  |
| **Metastatic sites** |  |  |  |  |  |  |  |  |  |
| **Lymph node** | 0 | 0 | inf | 1 |  |  |  |  |  |
| **Lung** | 0 | 0 | inf | 1 |  |  |  |  |  |
| **Liver** | 40.5 | 4.716 | 347.806 | ＜0.001* | 40.828 | 0.838 | 1989.268 | 0.061 |  |
| **Bone** | 0.127 | 0.021 | 0.765 | 0.024* | 6.87E+09 | 0 | inf | 0.999 |  |
| **Bone-only** | 0.311 | 0.054 | 1.805 | 0.193 |  |  |  |  |  |
| **Visceral-only** | 18.75 | 2.757 | 127.513 | 0.003* | 6.17E+09 | 0 | inf | 0.999 |  |
| **PIP mRNA** | 1.078 | 0.953 | 1.219 | 0.235 |  |  |  |  |  |

**Table S4** Univariate and multivariate logistic regression in CD45+ regions

| **Characteristics** | **Univariate analysis** | | | | **Multivariate analysis** | | | | |
| --- | --- | --- | --- | --- | --- | --- | --- | --- | --- |
|  | **OR** | **CI95% lower** | **CI95% upper** | **P value** | **OR** | **CI95% lower** | **CI95% upper** | **P value** |  |
| **Age** | 1.67 | 1.072 | 2.604 | 0.023* | 1.171 | 0.696 | 1.97 | 0.553 |  |
| **Histological grade** | 2.82E+13 | 0 | inf | 1 |  |  |  |  |  |
| **T stage** | 0.667 | 0.104 | 4.261 | 0.668 |  |  |  |  |  |
| **N stage** | 0.455 | 0.162 | 1.278 | 0.135 |  |  |  |  |  |
| **M stage** | 0 | 0 | inf | 1 |  |  |  |  |  |
| **ER status** | 12242786 | 0 | inf | 0.995 |  |  |  |  |  |
| **PR status** | 1.269 | 0.497 | 3.244 | 0.619 |  |  |  |  |  |
| **HER2 status** | 0.566 | 0.175 | 1.833 | 0.342 |  |  |  |  |  |
| **Metastatic sites** |  |  |  |  |  |  |  |  |  |
| **Lymph node** | 0 | 0 | inf | 1 |  |  |  |  |  |
| **Lung** | 0 | 0 | inf | 1 |  |  |  |  |  |
| **Liver** | 54 | 3.931 | 741.79 | 0.003* | 12.584 | 0.015 | 10492.21 | 0.460 |  |
| **Bone** | 0.2 | 0.03 | 1.317 | 0.094 | 52754895 | 0 | inf | 0.996 |  |
| **Bone-only** | 0.433 | 0.068 | 2.764 | 0.376 |  |  |  |  |  |
| **Visceral-only** | 12 | 1.623 | 88.702 | 0.015* | 31735114 | 0 | inf | 0.996 |  |
| **PIP mRNA** | 1.009 | 1 | 1.018 | 0.044* | 1.005 | 0.994 | 1.016 | 0.350 |  |

**Table S5** Univariate and multivariate logistic regression in PANCK+ regions

**Table S6** Univariate and multivariate logistic regression in CD45−/PANCK− regions

| **Characteristics** | **Univariate analysis** | | | | **Multivariate analysis** | | | | |
| --- | --- | --- | --- | --- | --- | --- | --- | --- | --- |
|  | **OR** | **CI95% lower** | **CI95% upper** | **P value** | **OR** | **CI95% lower** | **CI95% upper** | **P value** |  |
| **Age** | 1.813 | 1.117 | 2.943 | 0.016* | 1.472 | 0.776 | 2.793 | 0.236 |  |
| **Histological grade** | 3.47E+09 | 0 | inf | 0.997 |  |  |  |  |  |
| **T stage** | 0.5 | 0.086 | 2.904 | 0.440 |  |  |  |  |  |
| **N stage** | 0.672 | 0.299 | 1.509 | 0.335 |  |  |  |  |  |
| **M stage** | 0 | 0 | inf | 1 |  |  |  |  |  |
| **ER status** | 0.75 | 0.12 | 4.691 | 0.758 |  |  |  |  |  |
| **PR status** | 0.936 | 0.431 | 2.032 | 0.867 |  |  |  |  |  |
| **HER2 status** | 0.526 | 0.177 | 1.57 | 0.250 |  |  |  |  |  |
| **Metastatic sites** |  |  |  |  |  |  |  |  |  |
| **Lymph node** | 0 | 0 | inf | 1 |  |  |  |  |  |
| **Lung** | 0 | 0 | inf | 1 |  |  |  |  |  |
| **Liver** | 42 | 4.896 | 360.262 | ＜0.001* | 60.168 | 0.144 | 25182.46 | 0.183 |  |
| **Bone** | 0.121 | 0.02 | 0.728 | 0.021* | 1.61E+16 | 0 | inf | 1 |  |
| **Bone-only** | 0.292 | 0.05 | 1.685 | 0.168 |  |  |  |  |  |
| **Visceral-only** | 19.5 | 2.872 | 132.41 | 0.002* | 1.48E+16 | 0 | inf | 1 |  |
| **PIP mRNA** | 1.269 | 1.051 | 1.532 | 0.013* | 1.119 | 0.826 | 1.515 | 0.468 |  |

**Table S7** Univariate and multivariate Cox regression in CD45+ regions

| **Characteristics** | **Univariate analysis** | | | | **Multivariate analysis** | | | | |
| --- | --- | --- | --- | --- | --- | --- | --- | --- | --- |
|  | **HR** | **CI95% lower** | **CI95% upper** | **Overall P value** | **HR** | **CI95% lower** | **CI95% upper** | **Overall P value** |  |
| **Age** | 1.021 | 0.964 | 1.08 | 0.484 |  |  |  |  |  |
| **Histological grade** |  |  |  | 0.128 |  |  |  |  |  |
| **T stage** | 1.25 | 0.63 | 2.46 | 0.528 |  |  |  |  |  |
| **N stage** |  |  |  | 0.796 |  |  |  |  |  |
| **M stage** | 1.2 | 0.28 | 5.12 | 0.807 |  |  |  |  |  |
| **ER status** |  |  |  | 0.04* |  |  |  | 0.158 |  |
| **PR status** |  |  |  | 0.004* |  |  |  | 0.025* |  |
| **HER2 status** |  |  |  | 0.104 |  |  |  |  |  |
| **Metastatic sites** |  |  |  |  |  |  |  |  |  |
| **Lymph node** | 0.56 | 0.23 | 1.35 | 0.195 |  |  |  |  |  |
| **Lung** | 0.48 | 0.2 | 1.18 | 0.11 |  |  |  |  |  |
| **Liver** | 2.18 | 1.022 | 4.67 | 0.044* | 2.47 | 0.6 | 10.25 | 0.212 |  |
| **Bone** | 0.58 | 0.29 | 1.14 | 0.113 |  |  |  |  |  |
| **Bone-only** | 1.52 | 0.77 | 2.99 | 0.225 |  |  |  |  |  |
| **Visceral-only** | 2.04 | 0.974 | 4.27 | 0.059 | 2.95 | 1.021 | 8.54 | 0.046* |  |
| **PIP mRNA** | 1.006 | 1 | 1.012 | 0.038* | 1 | 0.993 | 1.008 | 0.968 |  |

**Table S8** Univariate and multivariate Cox regression in PANCK+ regions

| **Characteristics** | **Univariate analysis** | | | | **Multivariate analysis** | | | | |
| --- | --- | --- | --- | --- | --- | --- | --- | --- | --- |
|  | **HR** | **CI95% lower** | **CI95% upper** | **Overall P value** | **HR** | **CI95% lower** | **CI95% upper** | **Overall P value** |  |
| **Age** | 1.013 | 0.953 | 1.08 | 0.675 |  |  |  |  |  |
| **Histological grade** |  |  |  | 0.234 |  |  |  |  |  |
| **T stage** | 1.44 | 0.71 | 2.92 | 0.309 |  |  |  |  |  |
| **N stage** |  |  |  | 0.877 |  |  |  |  |  |
| **M stage** | 1.33 | 0.31 | 5.71 | 0.705 |  |  |  |  |  |
| **ER status** |  |  |  | 0.256 |  |  |  |  |  |
| **PR status** |  |  |  | 0.015* |  |  |  | 0.021* |  |
| **HER2 status** |  |  |  | 0.105 |  |  |  |  |  |
| **Metastatic sites** |  |  |  |  |  |  |  |  |  |
| **Lymph node** | 0.6 | 0.24 | 1.47 | 0.264 |  |  |  |  |  |
| **Lung** | 0.52 | 0.21 | 1.27 | 0.15 |  |  |  |  |  |
| **Liver** | 2.17 | 0.92 | 5.15 | 0.078 | 1.9 | 0.61 | 5.84 | 0.266 |  |
| **Bone** | 0.63 | 0.31 | 1.28 | 0.196 |  |  |  |  |  |
| **Bone-only** | 1.63 | 0.8 | 3.29 | 0.177 |  |  |  |  |  |
| **Visceral-only** | 1.72 | 0.77 | 3.86 | 0.185 |  |  |  |  |  |
| **PIP mRNA** | 1.18 | 0.96 | 1.45 | 0.118 |  |  |  |  |  |

**Table S9** Univariate and multivariate Cox regression in CD45−/PANCK− regions

| **Characteristics** | **Univariate analysis** | | | | **Multivariate analysis** | | | | |
| --- | --- | --- | --- | --- | --- | --- | --- | --- | --- |
|  | **HR** | **CI95% lower** | **CI95% upper** | **Overall P value** | **HR** | **CI95% lower** | **CI95% upper** | **Overall P value** |  |
| **Age** | 1.012 | 0.955 | 1.07 | 0.688 |  |  |  |  |  |
| **Histological grade** |  |  |  | 0.383 |  |  |  |  |  |
| **T stage** | 1.36 | 0.68 | 2.71 | 0.378 |  |  |  |  |  |
| **N stage** |  |  |  | 0.851 |  |  |  |  |  |
| **M stage** | 1.33 | 0.31 | 5.72 | 0.699 |  |  |  |  |  |
| **ER status** |  |  |  | 0.165 |  |  |  |  |  |
| **PR status** |  |  |  | 0.002* |  |  |  | 0.007* |  |
| **HER2 status** |  |  |  | 0.079 |  |  |  | 0.002* |  |
| **Metastatic sites** |  |  |  |  |  |  |  |  |  |
| **Lymph node** | 0.6 | 0.25 | 1.47 | 0.266 |  |  |  |  |  |
| **Lung** | 0.51 | 0.21 | 1.25 | 0.14 |  |  |  |  |  |
| **Liver** | 2.31 | 1.08 | 4.97 | 0.032* | 7.96 | 1.63 | 38.96 | 0.01* |  |
| **Bone** | 0.52 | 0.26 | 1.032 | 0.061 | 0.63 | 0.14 | 2.81 | 0.542 |  |
| **Bone-only** | 1.38 | 0.7 | 2.73 | 0.358 |  |  |  |  |  |
| **Visceral-only** | 2.14 | 1.014 | 4.5 | 0.046* | 4.16 | 0.69 | 25.27 | 0.121 |  |
| **PIP mRNA** | 1.004 | 1.001 | 1.007 | 0.019* | 0.999 | 0.994 | 1.005 | 0.848 |  |
